# Prostate Cancer: Etiology, Diagnosis, and Treatment

**DOI:** 10.17161/sjm.v3i1.24992

**Published:** 2026

**Authors:** Chaohao Li, Zhiguo Li, Xiaoqi Liu

**Affiliations:** Department of Toxicology & Cancer Biology, The University of Kentucky College of Medicine, Lexington, KY 40536, USA

**Keywords:** prostate cancer, etiology, diagnosis, treatment

## Abstract

Based on cancer statistics in 2025, 313,780 American men were diagnosed with prostate cancer, and 35,770 of them died from the disease; thus, prostate cancer remains a serious health issue for American men. In this article, we review multiple aspects of the disease, including etiology, diagnosis, and treatment. Regarding disease etiology, we summarize current knowledge of prostate cancer initiation and progression, with a focus on androgen receptor signaling. We discuss the genetic landscape of prostate cancer, including alterations in the DNA damage response pathway, FOXA1, SPOP, the PI3K pathway, WNT signaling, AURKA, and MYCN. We also address the potential contribution of inflammation to prostate cancer development. In the diagnosis of prostate cancer, the Gleason score is discussed. Measurement of prostate-specific antigen (PSA) levels has been the gold standard for diagnosing prostate cancer and monitoring disease progression. We also review additional diagnostic methods, including magnetic resonance imaging, the 4Kscore, the Prostate Health Index, and bone scintigraphy. Various treatments have been used clinically. For localized prostate cancer, active surveillance, radical prostatectomy, radiotherapy, and androgen deprivation therapy are available options. For metastatic prostate cancer, androgen deprivation therapy remains the standard of care. For castration-resistant prostate cancer, cabazitaxel and second-generation androgen receptor inhibitors are the major therapeutic approaches. We also review several non-canonical treatment strategies.

## 1. Introduction

According to the cancer statistics of 2024, prostate cancer (PCa) continues to rank as the third most diagnosed type of cancer and the sixth leading cause of death [1]. As a disease that is closely related to aging, PCa tends to happen in older men [2]. In addition, other determinants such as ethnicity, family history, and hereditary changes of the genome are also implicated in PCa [3]. Androgen receptor (AR) signaling, which is the well-established pathway to sustain PCa’s survival and proliferation [4, 5], is essential for disease progression. Accordingly, medical interventions that deplete endogenous androgen, named androgen-deprivation therapy (ADT), remain the major treatment against PCa [6, 7]. Despite the slow progression of this disease, nearly all patients eventually progress to the deadly metastatic castration-resistant prostate cancer (CRPC) that is resistant to ADT. Significant efforts have been put into dealing with this situation, and that leads to the modification of current therapy regimens and the discovery of novel therapeutic agents.

In the past decades, significant breakthroughs have been made in this field, especially the discoveries of highly selective therapeutic agents targeting PCa vulnerabilities. These unparalleled advances were achieved through the large-scale screening of genomic data using whole-genome DNA sequencing, mRNA sequencing, and proteome profiling, which have delivered unique insights into the molecular contents underpinning different subtypes and the nature of PCa [8, 9]. However, we are still facing new challenges to be solved. Recently, as the immune checkpoint inhibitors have been successful in multiple cancers, immunotherapy in PCa is being intensively investigated [10]. Unfortunately, as one of the “cold tumors”, PCa is notorious for its weak induction of immune response and a profound immunosuppressive microenvironment [11]. Further study will need to unravel the molecular mechanism of the immunosuppressive milieu of PCa, such as the infiltration of immunosuppressive cells and the release of cytokines that lead to this unfavorable outcome.

Indeed, research about PCa is a multidisciplinary area that incorporates computational biology, laboratory, and clinical testing. Before the final application of a certain practice, a series of steps that validate preclinical hypotheses and scientific findings must be done to successfully translate them into clinical scenarios. These rigorous procedures are critical to improve disease management. As our knowledge about this disease continues to grow, improvement in early detection and treatment of the disease will finally transform the pattern of clinical care and benefit patients’ survival. This review will briefly summarize the current understanding of PCa, with a focus on the carcinogenesis, diagnosis, and treatment of this disease. Finally, the future direction of PCa research will be discussed.

## 2. Disease Etiology

**Figure 1.**
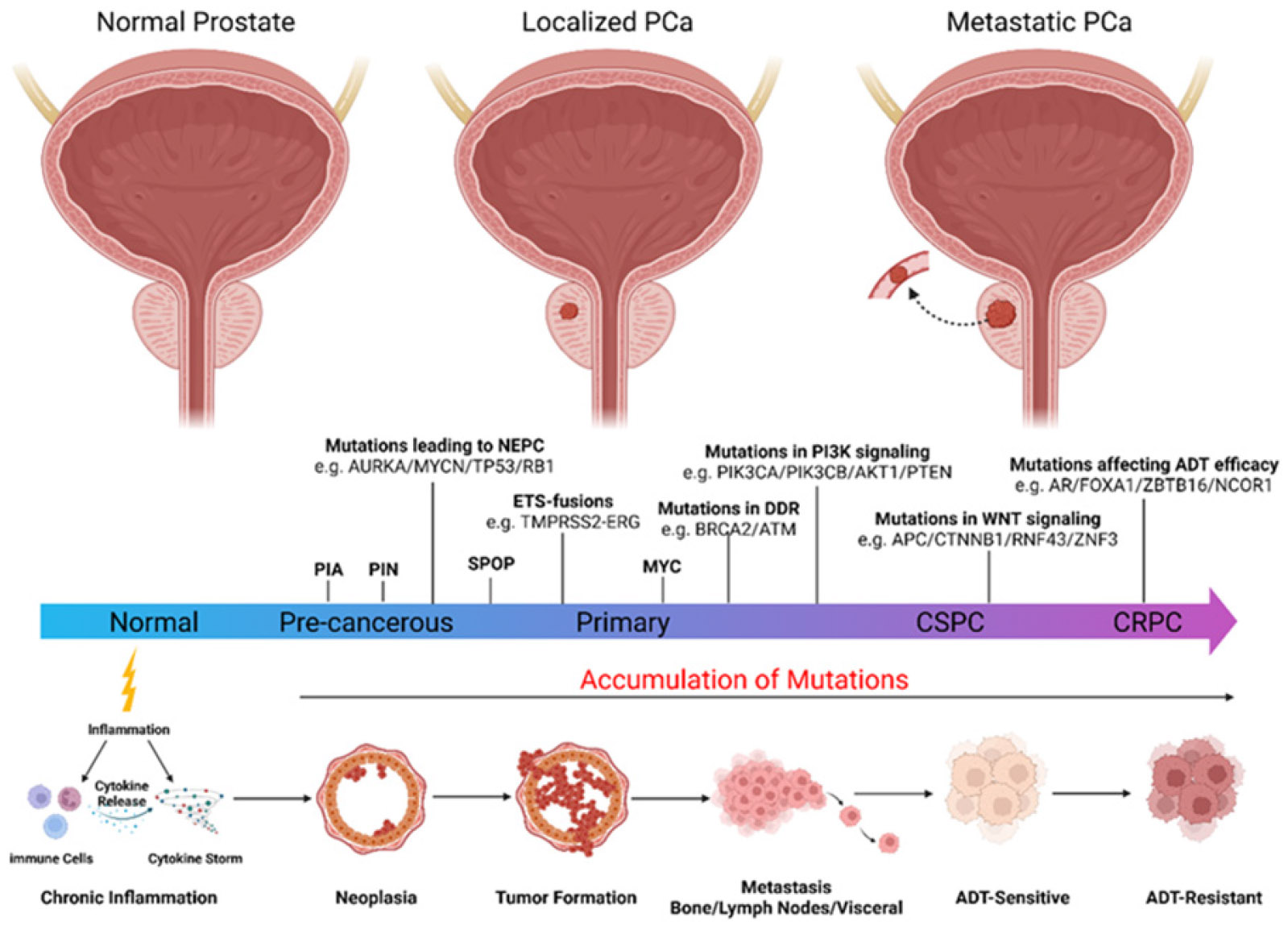
Prostate carcinogenesis. PCa is thought to be driven by the accumulation of harmful mutations. Inflammatory events triggered by either external or internal stimuli are the early sin of carcinogenesis. Marked by the infiltration of multiple types of immune cells, these cells will release cytokines, leading to cytokine storm and genetic instability. Certain pioneer mutations will cause abnormal proliferation of prostate tissue, termed PIA or PIN. Those pre-cancerous stages will eventually develop to primary tumor. Further mutations promote the progression and formation of metastatic PCa. Initially, metastatic PCa is sensitive to ADT, termed castration-sensitive PCa (CSPC). After a period, PCa is resistant to ADT, termed CRPC. It is believed that mutations affecting AR pathways are the key determinant of this transition. Created with BioRender.com.

The carcinogenesis of PCa is complicated and has not been completely understood yet. However, it is generally accepted that multiple pathological events are involved in this malignant change (Fig. 1). Accumulation of mutations is the ultimate driver of disease initiation and progression, and inflammation is thought to promote this process.

### 2.1 Initiation and Progression

The prostate is a reproductive organ consisting of three zones: the central layer, transition layer, and the peripheral layer [12, 13]. While cells in all zones have the capacity to develop cancer, the peripheral layer is the principal region of prostate neoplasms and carcinogenesis, making up about 75% of all disease cases [14]. Morphologically, the major structures of the normal prostate are ducts and acini, with stroma embedded in the entity [15]. Like other organs with ductal structure, the ducts of the prostate contain luminal cells and basal cells that form the basement membrane. Inside the basement membrane is the extracellular matrix and stromal cells, including smooth muscle myocytes and fibroblasts [16]. The luminal cells are generally accepted as the origin of PCa [17-19]. However, this theory has been questioned by increasing evidence supporting the basal origin of PCa [20-22]. These facts lead to the current hypothesis that both luminal and basal cell types are capable of giving rise to PCa, as supported by other studies [23, 24]. No matter what the origin of the PCa, it is clear that the transformation process must encompass several malignant changes to confer invasive phenotypes to the original cells, which then trigger uncontrolled proliferation and breakthrough of the boundary of the basement membrane. Usually, this process is first observable as proliferative inflammatory atrophy or prostate intraepithelial neoplasia, both of which are considered precursors of PCa and may eventually transform to malignant tumors in the future [25, 26]. In contrast, benign prostatic hyperplasia, another disease of the prostate that is usually associated with aging, does not provide prognostic value for PCa, in which only non-malignant cell proliferation happens in the transition zone of the prostate [27].

**Figure 2.**
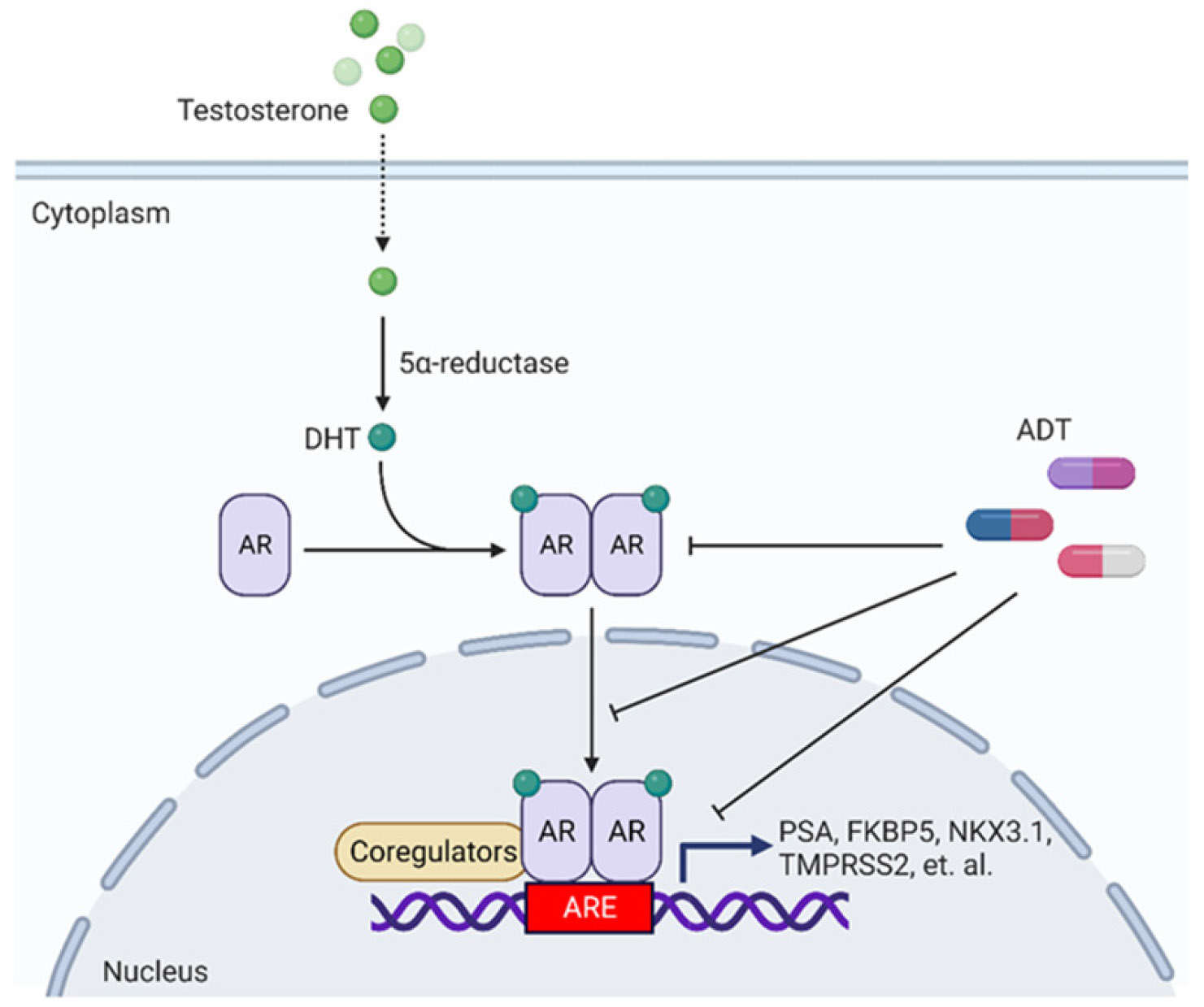
AR pathway and ADT. PCa is strongly associated with the AR pathway. The major androgen in serum is testosterone. It is often converted to dihydrotestosterone (DHT) by 5α-reductase, which is a more potent form of androgen that binds to AR. Without a ligand, AR is mainly located at cytoplasm. Following androgen ligand binding, active AR translocates to the nucleus and forms dimers, where it binds to androgen-response elements (AREs) and initiates the transcription of downstream targets. ADT specifically blocks AR pathways through multiple mechanisms, including inhibition of ligand binding, AR translocation, and the final transcription step. Adapted from “Androgen Receptor Genomic Pathway”, by BioRender.com (2022). Retrieved from https://app.biorender.com/biorender-templates.

The early onset of PCa accompanies a series of symptoms, such as urethral stricture, and routine screening of PCa for older people has led to most patients diagnosed at an early stage [28]. At this stage, PCa is curable with surgery or radiation therapy. Those who are unlikely benefit from curative treatment due to metastatic disease can be well-managed for a long time. AR is the most important target at this stage due to its dominant role in the survival of PCa. Without the binding of its ligand, androgens, AR is predominantly located in the cytoplasm. Upon binding to androgens, AR translocates to the nucleus where it binds to the androgen response element of its target genes to initiate transcription and drive tumorigenesis (Fig. 2). This is the basis of ADT, which aims to deplete endogenous androgens to control disease progression. At the beginning, patients respond well to ADT, so the disease is termed castration-sensitive PCa (CSPC). However, even though early diagnosis of PCa has a good prognosis under current management, a substantial portion of patients will experience biochemical recurrence or disease progression, which is usually concomitant with an elevated AR activity and serum prostate-specific antigen (PSA) levels, making patients unresponsive to ADT and thus termed CRPC. Progression to CRPC is frequently marked with visible metastasis to bone marrow due to the bone tropism of PCa [29, 30], and this event is the major cause leading to 90% cancer death [31]. Major steps of metastasis include detachment from cell-cell and cell–matrix contact, invasion and intravasation into the nearby blood vessels, survival through the physical force and immune system in the circulation system, extravasation to reside at the new site, and finally formation of metastatic lesions [32]. This is a multi-step process that is poorly understood [33]. Most often, cells must possess the ability to digest extracellular matrix, which is achieved by secretion of matrix metalloproteinases, and evade the immune system all the time, which might be achieved through the immune-suppressive feature of PCa [34]. Newly formed metastatic CRPC marks the failure of ADT and leads to SREs and symptoms, including bone pain, bone fractures, and spinal cord compression [35]. These complications negatively affect the normal bone remodeling cycle and patients’ quality of life. The metastatic event of PCa is a big issue, not only because of the bad outcome associated with it, but also the bad impact on the prognosis of treatment. Since occult metastases might be undetected at diagnosis, patients with invisible lesions will unavoidably experience therapeutic failure [36]. As a result, how to effectively detect occult metastases is under investigation.

### 2.2 Genetic Landscape

Accumulation of harmful mutations over the long term is thought to account for the carcinogenesis of PCa. These alternations of normal genomes, either in oncogenes or tumor suppressors, gradually render malignant phenotypes to normal prostate cells and slowly transform them into cancerous counterparts. According to the TCGA database, mutations mainly affect cell proliferation and death pathways, as well as the DNA damage response (DDR) pathways, which might be further explored for targeted therapies [37, 38]. Of note, none of them can distinguish aggressive PCa from indolent ones, which suggests that the onset of PCa is not owing to a dominant driver. Instead, large-scale genomic alterations are prognostic features of non-indolent PCa, suggesting that the progression of PCa is due to the joint efforts of multiple mutations [39]. Indeed, very few genes are targetable, and this is a big challenge for early disease management. Hence, a deeper understanding of the genomic profile will likely guide the treatment strategies of PCa with different features and prognosis.

The mutation landscapes among various stages of PCa display a significant difference. In patients with localized PCa, gene fusions between family members of ETS transcription factors and AR-targeted genes are seen in more than 50% of localized PCa [40, 41], of which the most common one is the fusion of ERG with TMPRSS2. Whole-genome sequencing of the primary tumor also indicates other minor mutations that deserve attention, including the loss-of-function mutations in E3 ligase SPOP and gain-of-function mutations in transcription factor FOXA1 [42]. Increasing evidence suggests that mutations of these two genes have a predominant role in the disease progression. As an E3 ligase, impaired function of SPOP promotes the stabilization of many oncogenic proteins and rewiring of downstream pathways, such as DEK, SRC3, and BRD4 [43-45]. FOXA1 is an AR cofactor that increases the transcriptional ability of AR to promote cancer progression [46, 47]. Furthermore, these mutations could jointly lead to progression by interacting with each other. For instance, it has been reported that fusions of ERG with TMPRSS2 are resistant to SPOP-mediated degradation [48]. Considering the tumor-promoting effect of SPOP mutation, the co-occurrence of two distinct mutations may further increase the likelihood of disease progression and recurrence after treatment. Future studies will need to address similar questions by evaluating the predictive values of a single prominent mutation or its combinations.

Progression from localized PCa to metastatic PCa is coupled with deregulation of several genes. Alterations of the PI3K pathway are frequently seen with gain-of-function mutations in PIK3CA, PIK-3CB, and the downstream kinase AKT1 [49, 50]. PTEN, the phosphatase that negatively regulates the PI3K pathway, also frequently encounters deletion and loss-of-function mutations, which are displayed in about 20% of primary tumors but increase to 50% in CRPC [51]. Another important event is the activation of the WNT signaling pathway in CRPC, which may account for the disease progression [52]. This happens as the mutations of APC and CTNNB1 that directly activate canonical WNT signaling, which are observed in about 20% of CRPC patients [53], or mutations in inhibitory components such as RNF43 and ZNRF3 [54]. Besides, activation of noncanonical WNT signaling is also observed in CRPC, which deserves further exploration [55]. The well-known oncogene c-Myc is also implicated in PCa progression [56]. c-Myc is highly expressed at every stage of PCa through regulations by other genes, which itself stimulates an embryonic stem cell-like signature to drive proliferation and therapy resistance [57, 58]. These significant mutation events in disease progression deserve further attention, and clinical trials to test potential drug candidates that targeting aforementioned pathways should considered in the future.

In CRPC, perhaps the most common genetic alterations are around AR. This important target in PCa undergoes various types of mutations. Mutations most often happen in the form of amplification or gain-of-function mutations of AR per se, but can also involve its associated co-activators and repressors. FOXA1 is the confirmed co-activator of AR related to progression to CRPC [59]. In addition, inactivating mutations or deletions of repressors of AR transcriptional activity, such as ZBTB16 and NCOR1, are detected in substantial portions of CRPC patients [60, 61]. This is in contradistinction to CSPC patients, in which mutations of AR are fairly uncommon [62]. This phenomenon suggests that mutations of AR are the driving force of progression to CRPC and key determinants of resistance to ADT. Overexpression of AR through gene amplification is both necessary and sufficient to confer PCa resistance to ADT. Mutations in the ligand-binding domain of AR can lead to changes in ligand selectivity, resulting in an antagonist-agonist switch of drugs or promiscuity of ligand binding that enables activation by other structurally similar hormones [63-65]. Alternative splicing of the AR gene results in AR splice variants that remain constitutively active. These AR isoforms lack the ligand-binding domains, so they are not affected by AR-target therapies and contribute to resistance to ADT in clinical situations [66-69]. Considering the importance of AR throughout disease progression, ADT is commonly continued on a lifelong basis, but further understanding of genetic alterations driving resistance to ADT is critical for the long-term efficacy of this therapy.

Another class of mutations is genes involved in DDR, which is a hallmark of metastatic PCa compared to localized disease [70]. Mutations happen in about 10% of metastatic PCa cases, and the most prevalent mutations are BRCA2 and ATM, which are involved in homologous recombinant repair of double-strand breaks [71]. Other minor alterations are those involved in mismatch repair (such as MSH2 and MSH6) and nucleotide excision repair (such as ERCC2 and ERCC5). Interestingly, metastatic PCa tends to have a higher mutation burden than localized PCa [72], and AR has been reported to regulate DDR genes [73]. Considering that most biopsy samples were obtained from CRPC patients who had been treated with ADT, the higher mutation burden in the late stage of this disease may reflect the synergistic effect of ADT and DDR mutations. Furthermore, mutations seem to be enriched in CRPC compared to CSPC, indicating that this class of mutations has an important role in promoting disease progression. Of note, cells with defects in the homologous repair pathway deficiency result in increased sensitivity to PARP inhibitors, which is termed synthetic lethality [74, 75]. The idea of synthetic lethality has been successfully applied in the treatment of breast cancer with BRCA2/BRCA1 mutations, and this leads to the motivation to investigate whether this subset of PCa patients may respond to PARP inhibitors. Several large-scale clinical trials are ongoing to test this possibility.

Certain genes may uniquely mark a rare variant of PCa. This subset of PCa morphologically mimics the small cell carcinoma and lacks AR expression. Instead, they are enriched with neuroendocrine markers, including chromogranin A, synaptophysin, and neuron-specific enolase [76]. Thus, they are termed neuroendocrine PCa (NEPC). The most frequent mutations in these tumors are amplifications of AURKA and MYCN [77]. Besides, TP53 and RB1 are frequently mutated in NEPC, which concordantly promote lineage plasticity, metastasis, and resistance to ADT [78-80]. De novo NEPC only accounts for a small subset of all PCa. However, since ADT is the standard treatment for PCa, cells with NEPC features are resistant to ADT and may display a survival advantage over bulky tumors, resulting in selective enrichment of this population and treatment-induced NEPC [81]. Currently, the precise molecular profile of treatment-induced NEPC is still not clear, but the trans-differentiation process is likely involved in this transition [82]. Other possible mechanisms, such as hypoxia signaling or epigenetics, need further validation [83, 84]. Sadly, because of its aggressive phenotype, very limited treatment methods are available for NEPC. Therefore, more investigations to develop treatment options should be guaranteed in the future.

### 2.3 Inflammation

Although the precise etiology of PCa is not completely discerned, chronic and repeated inflammation is believed to drive PCa in the long term [85-87]. Due to its specific physiological location and frequent exposure to multiple stimuli, the prostate is susceptible to inflammation, making prostatitis very common among the male population [86]. Of note, major inducers of prostatitis such as bacterial infection, chemical exposure, and obesity have been identified as risk factors of PCa [88-93]. Chronic and persistent inflammation significantly damages the normal epithelial barrier of the prostate. The repair of the epithelial barrier results in PIA or PIN, both of which are considered the pre-cancerous condition of PCa and may transform to malignant tumors in the future [25, 26]. Thus, a deep understanding of the interplay between inflammation and PCa is required to establish the disease model. This will also provide insights into pathogenesis and early prevention of PCa.

While the causes of inflammation and prostatitis are disparate among different people, they all negatively impact the prostate tissue homeostasis in multiple aspects. Inflammation is marked by the infiltration of immune cells and the release of cytokines. These cells and cytokines significantly affect prostate carcinogenesis and disease severity. Upon being recruited to prostate tissue, immune cells will release various pro-inflammatory cytokines to promote the transformation of normal prostate cells. On the one hand, active immune cells often secrete free radicals, which can damage the DNA and cause genetic mutations correlated with the initiation of PCa [94-96]. Cells that harbor certain mutations possess a significant survival advantage over other populations under the inflammatory condition. For instance, loss of tumor suppressor NKX3.1 during inflammation is an early event of tumorigenesis, which dramatically accelerates the progression of PCa and associates with higher Gleason score (GS) [97-99]. Another important tumor suppressor, PTEN, is frequently lost during inflammation, which then potentiates the CXCL8 signaling to sustain the growth and survival of prostate epithelium [100]. Indeed, there are certain links between genetic mutations of PCa and the associated inflammatory condition. However, a thorough understanding of this relationship is still largely unknown. Further investigations will need to establish the correlation between these two important early events during prostate carcinogenesis.

Inflammatory signaling also serves as an accomplice to promote cancer progression, and this is due to the interplay between cytokines and other oncogenic pathways [101]. Common inflammatory cytokines IL-1β, IL-6, IL-8, TNF-α, and IFNγ are all implicated in prostate cancer progression and associated with poor survival outcomes [102-106]. More importantly, the interaction of some inflammatory signaling with the AR pathway is detrimental, as this interplay promotes progression to CRPC that is resistant to ADT. For example, elevated expression of IL-6 increases the nuclear translocation and DNA binding of AR, which is accompanied by activation of STAT3 and MAPK pathways. As a result, PCa can survive in an androgen-independent manner. Another cytokine, IL-23, which is released by myeloid-derived suppressive cells, can also enhance the activity of AR and sustain cell survival under androgen depletion conditions [107]. This convoluted interaction between inflammatory signaling and the AR pathway deserves our attention, as it may render novel methods to overcome drug resistance at the CRPC stage. So far, there is some evidence linking the cytokine signaling with the emergence of resistance to ADT [108, 109]. Further study will have to explore the possibility of targeting inflammatory signaling for PCa treatment.

## 3. Diagnosis of PCa

The prostate gland is located below the bladder and in front of the rectum. Since the urethra is surrounded by the prostate, most patients will suffer symptoms related to urination, such as dysuria and nocturia. When patients have these complications, and there is a high risk of PCa, a physician will have to investigate it. The standard diagnosis of PCa starts with a digital rectal exam, which is a physical palpation of prostate tissue to assess the condition. However, a digital rectal exam doesn’t provide any clues on the histological index of prostate, so prostate tissues are needed to evaluate the disease stage, which is achieved by transrectal or transperineal biopsy of prostate [110]. Pieces of tissue are examined by microscopy to see whether there is any pathological change. To stratify the disease severity, samples will usually be graded with a Gleason Score (GS), which conveys a number to samples based on the pattern of biopsy tissue [111]. A sample will be graded with two scores. The first score is used to evaluate the dominant pattern of tissue, while the second score is for the minority cell pattern. Both scores have a scale of 1 to 5, so the complete GS of a sample is the sum of the two scores. The higher the GS, the worse the disease and outcome. Currently, the Gleason grading system has been reorganized into the International Society of Urologic Pathologists grade system that separates GS into 5 categories [112, 113]. It denotes GS of 6 and 8 as Group 1 and Group 4, GS of 9 or 10 as Group 5. For GS of 7, it further differentiates GS of 3+4 from 4+3. Since the clinical evidence delineates that patients with GS of 4+3 have obvious worse prognosis [114, 115], patients with GS of 3+4 are defined as Group 2, and those with GS of 4+3 are classified into Group 3.

Another canonical diagnosis method is to measure molecular biomarkers of PCa. Since PCa relies on AR pathways to sustain survival and develop malignant phenotype, detection of serum PSA, which is the downstream target of AR, is another gold standard for the diagnosis of PCa. The introduction of PSA as a diagnostic tool marks an unprecedented event in the history of the PCa field and leads to a burst of patients diagnosed with this disease, especially for younger men who are at the early stage of PCa [116]. Since then, PSA has been widely used in the diagnosis of PCa and the prediction of patients’ outcomes. The level of PSA is proportional to the severity of PCa, which indicates that patients with higher PSA is more likely to develop late-stage PCa with a predilection to metastatic disease [117]. In addition, PSA can also be leveraged to predict the efficacy of treatment and the potential recurrence of PCa. Higher PSA level before radiation therapy implies a higher likelihood of treatment failure and mortality [118, 119]. After ADT, the nadir PSA level and time to reach it may help to predict the efficacy of ADT [120, 121]. Indeed, serum PSA is a good benchmark for the possibility of biochemical recurrence after radical prostatectomy [122-124]. Because of various advantages and the convenience of PSA detection, the American Cancer Society provides a guideline that those who are at risk of PCa should be tested for PSA regularly to detect early onset of disease.

Unfortunately, the established diagnosis methods have obvious drawbacks under certain circumstances. While prostate biopsy helps identify bona fide pathological lesions, it frequently avoids the area of PCa and provides a false-negative result [125]. Actually, the detection rate of PCa is fairly low, even though multiple biopsies can somehow improve the discovery rate [126]. A report finds that this method misses nearly one-fourth of all PCa cases and often mismatches the scores with actual disease severity [127]. Regarding the PSA detection, there is a debate about which cutoff value is optimal for clinical practice. A low threshold often results in the overdiagnosis and overtreatment of those indolent PCa that have minimal impact on life [128, 129]. In fact, some studies argue that the overdiagnosis rate of PSA screening can be as high as over 50% [130-132], which is an astonishing number deserving our attention. However, a high cutoff also leads to other potential issues. False-negative rate pops up when a high cutoff is set, as it misses potentially aggressive cases that, when later found, are hard to manage [133]. Besides, high cutoff also produces inferior tracking of disease burden in terms of treatment-induced AR-negative PCa, which accounts for nearly one third of all cases after ADT [134].

Considering the weakness of mainstream diagnosis tools, significant refinement should be made to improve the precision of diagnosis. The advancement of radiography has coined more powerful diagnostic technologies. For example, targeted biopsy, which utilizes multiparametric magnetic resonance imaging to locate potential lesions before biopsy [135], displays superiority over standard prostate biopsy in that it provides a competitive discovery rate of clinically significant PCa while ruling out those insignificant ones [136, 137]. Currently, we are in a predicament of how to point out those clinically insignificant PCa that don’t need medical intervention. Whether patients’ quality of life will deteriorate due to PCa depends on the aforementioned risk factors and comorbidities [138]. Thus, to avoid overdiagnosis and overtreatment, several PCa risk calculators are designed to help evaluate patients’ health conditions and the necessity of treatment [139, 140]. Although most of them still need optimization, these programmed estimators help to define disease from an unbiased viewpoint.

Novel diagnostic tools are emerging to address the accuracy of traditional methods, especially in the field of molecular biomarkers that identify PCa. Since the level of PSA is greatly affected by individual prostate volume and health condition, it lacks specificity and often delivers unreliable results. To refine this method, combinatory detection of multiple PSA proteins or related indices has been popular. For example, the 4Kscore and Prostate Health Index are two commercially available tests used for decision-making of biopsy and prediction of progression [141, 142]. Detection of PCa antigen 3 in urine samples resolves the issue of specificity for PCa cells and decreases the negative rate of follow-up biopsy [143]. Detection of some genes in biopsy tissues is useful to predict the malignancy of cancer and can be applied to distinguish patients who need intensive medication. For example, the assessment of the Cell Cycle Progression score (Prolaris), which consists of 31 genes, is a reliable tool for doctors to improve prognosis [144]. Representative genes of various pathways can pick out presumably malignant cases that need immediate treatment, despite tumor heterogeneity, multifocality, and limited sampling. Based on this notion, a 17-gene Genomic Prostate Score (Oncotype DX) improves the prediction of pathological outcome and provides a guideline for treatment [145]. The Decipher test, which is based on a 22-gene genomic classifier, has been validated in the clinic to predict early metastatic event after prostatectomy [146].

Huge advancement has also been made to increase the sensitivity of detecting metastatic PCa. Bone scintigraphy using technetium-99m methylene diphosphonate, together with computed tomography and magnetic resonance imaging, is the standard diagnostic tool for staging of metastatic PCa. However, the frequent misses of micro-metastases and occult metastases discourage its reliability [147]. To deal with this problem, several radiotracers have been tested in positron emission tomography [148]. Exemplary reagents, including ^11^C-choline, ^18^F-fluciclovine, ^18^F-sodium fluoride, and ^68^Ga-PSMA-11, are available for the detection of metastatic lesions in lymph nodes and bones [149-152]. As advanced techniques are being created, patients will eventually benefit from these more sensitive and specific diagnosis regimens.

## 4. Treatment for PCa

**Figure 3.**
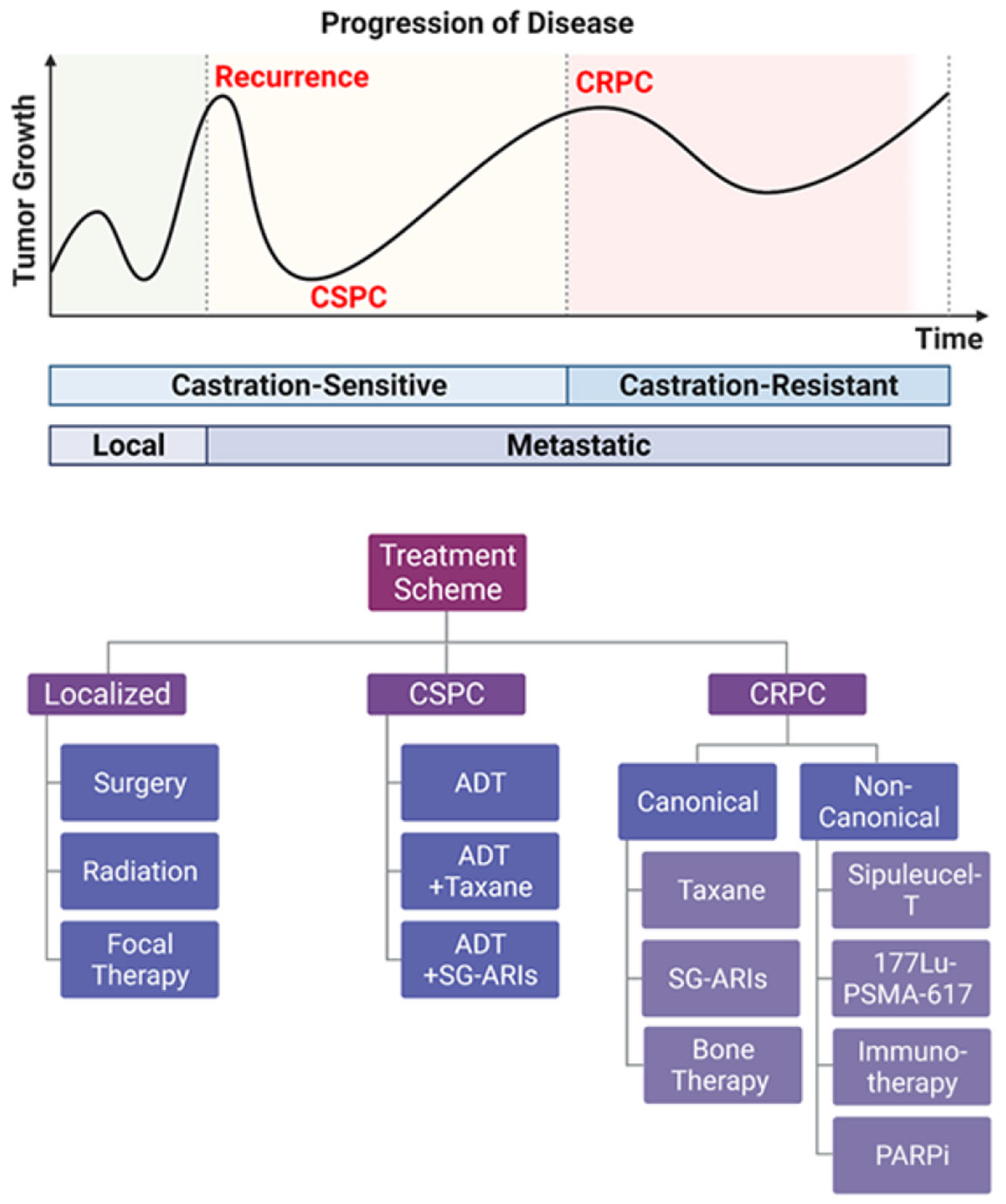
Treatment scheme for PCa. Treatment for PCa depends on the stage of the disease. For localized PCa, the disease is curative with surgery, radiation, or focal therapy, which is a more precise treatment targeting only tumor nodules. However, a substantial number of patients fail the treatment and develop biochemical recurrence accompanied by metastasis. At the CSPC stage, the current treatment modality has been modified, which is usually intensified with combination therapy of canonical ADT plus Taxane or SG-ARIs. However, progression to CRPC is still inevitable, and treatment approaches at this stage are almost the same, except for medicines targeting bone lesions. Some non-canonical treatment is available for a sub-cohort of patients, such as immunotherapy, 177Lu-PSMA-617, and PARP inhibitors. The only available medicine for NEPC is platinum-based therapy, which is not included in this figure. Adapted from “Natural History of Prostate Cancer” and “Flow Chart (5 Levels, Vertical) 6”, by BioRender.com (2022). Retrieved from https://app.biorender.com/biorender-templates.

Since the overdiagnosis and overtreatment of PCa are very common, whether PCa patients need medical intervention must be carefully deliberated. It should be noted that the 5-year survival of PCa is nearly 100%, and the risk of death not caused by PCa actually greatly exceeds death directly caused by the disease per se [153, 154]. Even a 29-year follow-up of PCa cases finds that merely one-third of all patients die of PCa, with life expectancy gained only 2.9 years in surgery treated groups [155]. In contrast, age and other risk factors are the leading causes of death, with patients carrying multiple comorbidities having the worst survivorship [156, 157]. Thus, treatment should be deferred unless other risk factors or comorbidities are identified that may negatively impact patients’ quality of life and overall survival. Life quality and patients’ acceptability should be taken into consideration before any treatment decision is made. Shared decision-making between patients and physicians should be the trend in the future [158]. Nonetheless, men with potentially aggressive cancer should receive treatment to control progression. Depending on the disease features at diagnosis, patients with localized cancer or metastatic cancer have different treatment options (Fig. 3).

### 4.1 Localized PCa

For patients with localized cancer, there are three treatment options: active surveillance, radical prostatectomy, or radiotherapy [5]. Generally, patients with low-risk PCa are well managed with active surveillance, and the chance of progression and metastasis is negligible [110]. Patients in this category will receive regular PSA screening and biopsy to monitor the disease status. Once a sign of progression is identified, such as a rise in PSA or upgrade of GS, active surveillance should stop, and regular treatment needs to be considered. However, there is an exclusion criterion for active surveillance in patients with low-risk PCa but combined with a cribriform or intraductal pattern of disease, which are associated with aggressive PCa and adverse outcomes [159, 160]. In that case, active surveillance should be avoided [161].

Patients with intermediate-risk PCa are not uniform and should be separated into two sub-categories [162, 163]. Low-intermediate patients tend to have a favorable outcome, so they can be provided with active surveillance after careful evaluation of other risk factors [164, 165]. However, for high-intermediate patients, active surveillance should be denied. Patients may choose to receive radical prostatectomy or radiotherapy combined with short-term ADT.

Patients with high-risk PCa are predisposed to malignant disease and metastasis, so they need immediate medical treatment. The choice is similar with those high-intermediate patients, but more intensive adjuvant therapy may be used to maximize efficacy. To avoid biochemical recurrence, detection of occult metastases is highly recommended before any treatment is applied. Surgery is performed to excise prostate tissue and associated pelvic lymph nodes. Depending on the post-operative PSA level, patients with detectable PSA may receive adjuvant therapy to remove residual tumors, while those with undetectable PSA levels are not recommended to receive further treatment [166, 167]. Instead, salvage radiotherapy is a potential curative approach for those with detectable PSA after surgery [168-170]. If radiotherapy is chosen, a long-term ADT is added to minimize biochemical recurrence. There is no evidence to demonstrate the obvious superiority of one method over another, but patients receiving different therapies may encounter divergent adverse effects. Of note, radiation therapy causes more nocturia and bowel dysfunction compared with radical prostatectomy, which tends worse urinary control and erectile function [171, 172].

Despite the curative outcome of local therapy, the subsequent sequelae may significantly downgrade patients’ lives. To address this issue, a more precise approach, dubbed focal therapy, is available for a small subgroup of patients with intermediate-risk, targetable volume of cancer [173]. Focal therapy displays an advantage over conventional local therapy in that it aims to eliminate cancer lesions while keeping healthy tissues intact. Due to this non-invasive feature, focal therapy decreases the risk of adverse outcomes associated with local therapy, such as urinary incontinence and erectile dysfunction [174, 175]. While focal therapy is superior to local therapy, there is no official guideline regarding its application criteria and desired outcome. However, as novel treatment modalities are being added to the armamentarium, focal therapy may eventually be recognized as the standard care for PCa patients [176].

### 4.2 Metastatic PCa

For patients with metastatic PCa, curative treatment such as radiotherapy and surgery is no longer available. Instead, ADT is the standard care for these patients, which is either achieved by surgical orchiectomy or drugs that deplete or antagonize endogenous androgen. Canonical ADT for CSPC includes luteinizing hormone-releasing hormone analogues that block de novo androgen synthesis or antiandrogen drugs that directly inhibit the effect of androgen [177]. Common side effects include hot flashes, sexual dysfunction, SREs, anemia, and complex metabolic syndrome [178, 179]. Besides, there is an increasing risk of cardiovascular disease in those patients [180-183]. Cognitive problem is a rare side effect but cannot be omitted [184, 185]. To relieve the caveats of continuous ADT, intermittent ADT is an alternative treatment approach for those patients. Marked by cyclical replenishment of androgen in patients, intermittent ADT is supposed to achieve noninferior disease control while reducing ADT-associated adverse effects and enhancing patients’ quality of life [186]. Patient selection is crucial for the best results of intermittent ADT, and this needs to be thoroughly investigated before any decision is made.

**Figure 4.**
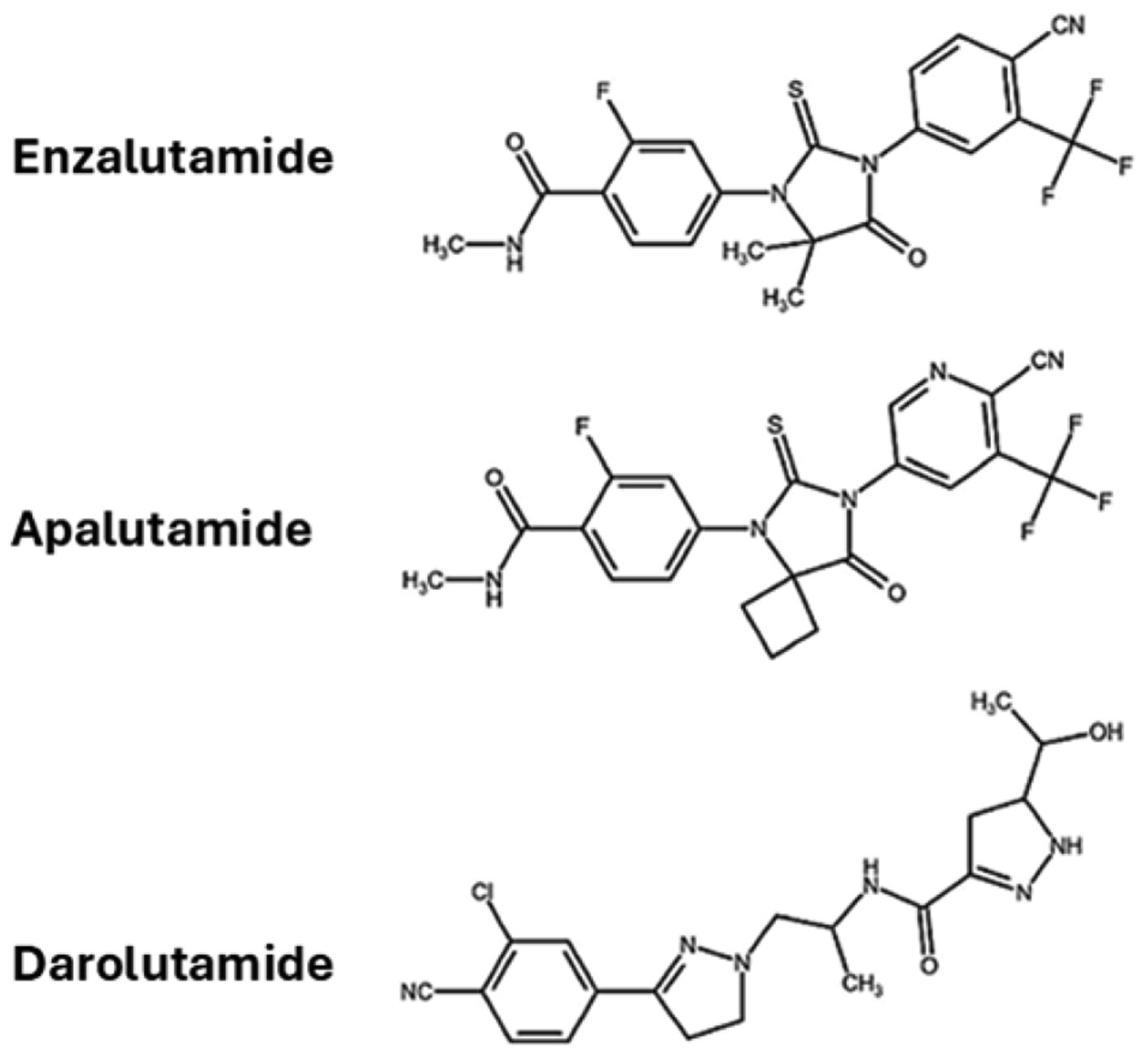
Structure of SG-ARIs. ENZ, APA, and DARO are three SG-ARIs used to treat CRPC patients. As illustrated, ENZ and APA share some structural similarity with similar backbones. However, DARO is distinct from ENZ and APA with some unique functional groups.

Unfortunately, nearly all primary treatments for metastatic PCa eventually fail, and the disease progresses to the CRPC stage. Over a long period, docetaxel is the standard treatment for CRPC [187]. For patients who progress after docetaxel treatment, cabazitaxel can provide survival benefits and delay disease progression [188]. However, both drugs only have a short-term effect, and cabazitaxel is not an ideal substitute for docetaxel as the first-line treatment [189]. To step out of this predicament, extensive research has been carried out to explore alternative treatments for CRPC. Evidence shows that AR continues to be the major driver of CRPC and thus remains a valid target [190]. Increased de novo androgen biosynthesis due to elevated CYP17A1 is one of the major reasons contributing to CRPC [191], and this finding has led to the application of CYP17A1 inhibitor abiraterone for the treatment of docetaxel-pretreated CRPC [192, 193]. Overexpression or amplification of AR is sufficient to induce CRPC and even alter the response nature of canonical ADT [194, 195]. Based on that, second-generation AR inhibitors (SG-ARIs) (Fig. 4), including Enzalutamide (ENZ), Apalutamide (APA), and Darolutamide (DARO), have emerged as a solution for CRPC patients after docetaxel treatment [196-198]. The emergence of SG-ARIs significantly improves patients’ survival. Of note, patients’ response to these drugs is somehow mutually exclusive, as indicated by the effectiveness of ENZ and abiraterone after docetaxel treatment, as well as the cabazitaxel after ENZ or abiraterone treatment [192, 193, 196, 199, 200]. This may be beneficial to patients, as different medicines can complement each other to deliver the optimal management of disease. To achieve the best disease control, the treatment sequence needs to be weighed before decision-making, especially when combining treatment strategies with patients’ characteristics. Besides, inherent or acquired drug resistance should be taken into consideration. Identification of molecular biomarkers associated with patients’ response before or during treatment will help design and modify treatment regimens.

While these drugs were previously solely used in CRPC, recent studies have demonstrated that early intensified medication with these drugs may provide benefits for disease control, as they may enhance patients’ response and delay the emergence of treatment resistance. Accordingly, the current regime of ADT for CSPC has been modified to a combination treatment with Taxane drugs or SG-ARIs. For example, docetaxel plus prednisolone is the standard treatment for CRPC, and the combination with canonical ADT for CSPC can prolong patients’ overall survival [201-203]. Likewise, a combination of abiraterone, ENZ, APA, or DARO with canonical ADT in CSPC also shows supremacy over single ADT in several clinical trials [204-209]. However, as all the studies were performed in an era when canonical ADT alone was used as a treatment for CSPC, whether these newly modified treatment strategies can benefit patients who previously received these agents for CSPC is not completely guaranteed, and more studies are needed to address this question.

**Figure 5.**
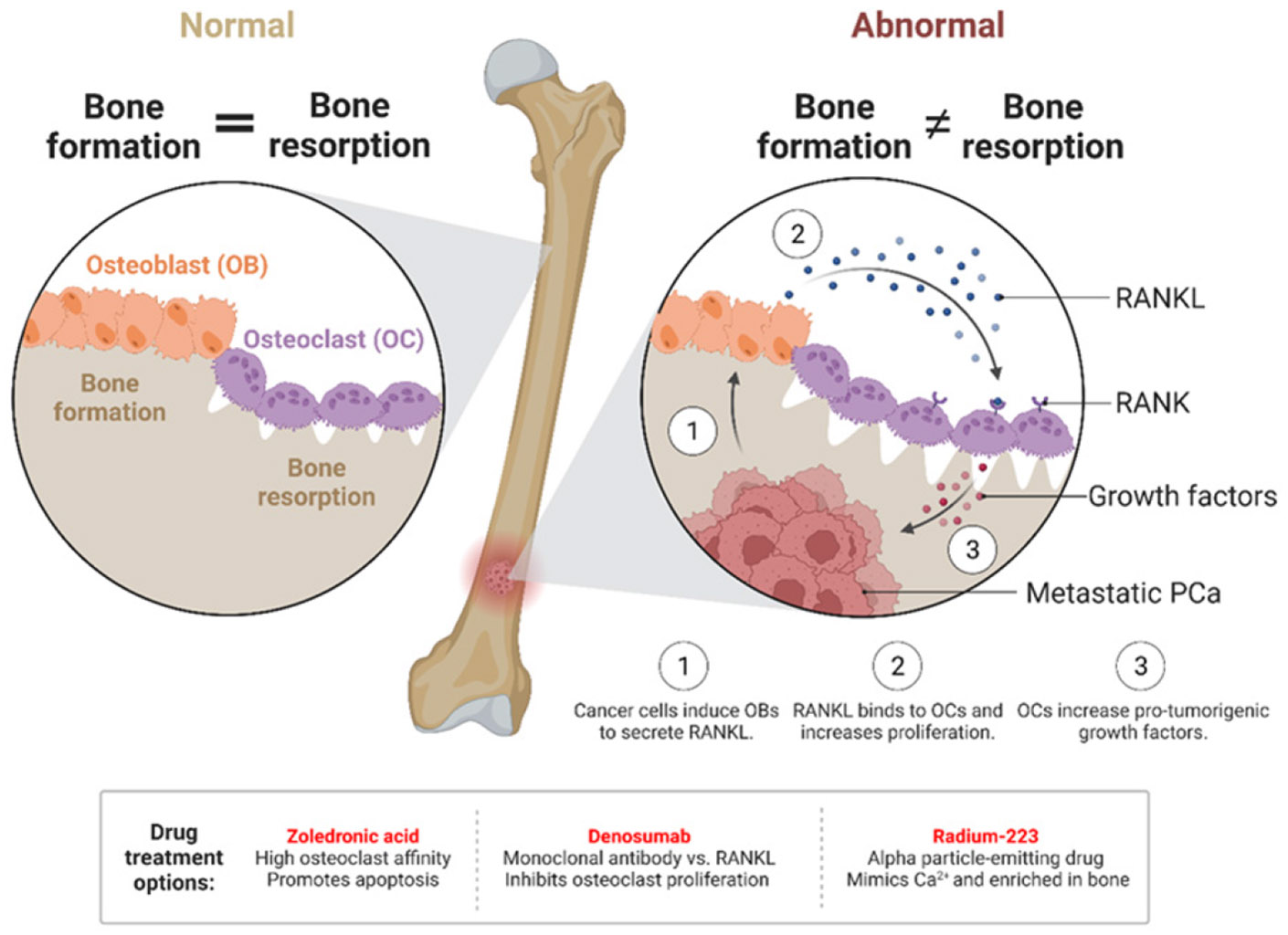
PCa bone metastasis and therapy. PCa has a strong preference for bone metastasis. Normal bone homeostasis is maintained by osteoblasts and osteoclasts, which are responsible for bone formation and resorption, respectively. Under pathological conditions of PCa metastasis, the balance of bone homeostasis is broken. This figure shows the typical osteolytic lesions, in which the bone resorption is enhanced over bone formation, although PCa can also cause osteoblastic lesions. The interaction between osteocytes and PCa promotes the proliferation of tumors and SREs in patients. Multiple therapeutic options are available for patients to relieve the symptoms. Adapted from “Metastasis to Bone Disrupts Bone Homeostasis”, by BioRender.com (2022). Retrieved from https://app.biorender.com/biorender-templates.

PCa has a high tendency to metastasize to bone. Once metastases have been identified, nearly 70% of PCa patients will develop bone metastases that are associated with SREs [210]. Because of this specific bone tropism of PCa, therapeutics that improve bone condition are an important component in the treatment regimen of metastatic PCa (Fig. 5). Since osteoclasts are the principal driver of bone effects, drugs that modulate the function of osteoclasts help to alleviate symptoms. Zoledronic acid is a canonical drug for PCa with bone metastases. However, the early administration doesn’t delay the onset of bone metastases [211]. A more recent medicine, called Denosumab, is favored to delay the onset of SREs by targeting the receptor activator of nuclear factor κ-B pathway, which is the key signaling in the maturation of osteoclasts [212, 213]. Besides, radiopharmaceutical radium-223 specifically targets the cancer cells residing in bone. Due to its chemical similarity to calcium, radium-223 is enriched in bone where it emits alpha particles to kill metastatic cancer cells. Clinical evidence shows that radium-223 elongates patients’ overall survival as well as palliates bony metastases-related symptoms [214]. So far, all medicines are only for palliative purposes, and there is still no treatment for preventing bone metastases of PCa. As our knowledge about PCa bone metastasis continues to grow, more options will be available to deal with this common complication of PCa.

### 4.3 Non-canonical Treatment

While the mainstream therapy for PCa is based on ADT and Taxane drugs, some non-canonical treatment options are available for a small subset of patients. Cancer cells recognized by immune cells are supposed to be eliminated, and this is the central rationale of immunotherapy. While the level of PSA is often used to monitor the activity of AR, it fails to be a good epitope for the identification of PCa cells, as the PSA-based vaccine doesn’t convey a survival benefit in PCa patients [215]. The failure of PSA as a valid target stimulates the exploration of other immune epitopes. One immunotherapy vaccine, called Sipuleucel-T, is based on the immunization against the prostatic acid phosphatase and elimination of cancer cells by T cells [216]. Phase 3 clinical trial showed that it could prolong patients’ overall survival for 4.1 months, which led to the FDA approval for asymptomatic or minimally symptomatic CRPC patients [217]. Recently, another promising approach has emerged that targets the membrane antigen PSMA of PCa cells. Directed by a cancer-specific PSMA epitope, radioactive compound lutetium-177 can destroy cancer cells with minimal side effects on adjacent tissues. This ^177^Lu-PSMA-617 radiopharmaceutical has been tested in phase 2 and phase 3 trials, and the results are very exciting. Compared to canonical ADT or Taxane-based therapies, ^177^Lu-PSMA displays superiority to improve overall survival and quality of life with good tolerance and low toxicity in CRPC patients [218, 219]. Based on these results, the FDA approved this treatment for CRPC on March 23rd, 2022.

As the genetic profiles of PCa patients are being profoundly interrogated, novel therapeutic options have emerged to specifically target the genomic vulnerabilities of cancer cells. The anti-PD-1 or anti-PD-L1 immunotherapy may work for a subset of heavily pretreated CRPC patients with mismatch repair deficiency, which is characterized by microsatel lite instability and high tumor mutation burden [220, 221]. Interestingly, the response to anti-PD-1 therapy seems to be unrelated to the expression of PD-L1 [222, 223]. However, this treatment modality fails to provide a substantial effect to control disease progression, as only a subset of patients will benefit from it. Due to this insufficiency, a combination of immune checkpoint inhibitors with docetaxel or ENZ might be a better choice and is still being investigated [224, 225]. Another subset of patients with loss of CDK12 may also benefit from immune checkpoint inhibition, due to the elevated genomic instability, neoantigen burden, and T cell infiltration [226]. Mutations of genes involved in homologous recombination repair consist of about a quarter of the total genetic alterations in CRPC [227, 228]. Among these genetic deficiencies, BRCA2/1 is one of the most frequently mutated genes, which makes up 12% of all lesions. PARP inhibitors that lead to synthetic lethality have been successfully administered in breast and ovarian cancers harboring BRCA2/1 mutations [75]. This inspires the application of PARP inhibitors in CRPC patients. Clinical trials of Olaparib and rucaparib show significant results to control disease progression [229, 230], which leads to the FDA approval of these to treat patients with BRCA2/1 mutations. Together with the tests for BRCA2/1, such as Foundation One and BRAC Analysis, these potent therapeutic approaches further improve the medical care for late-stage patients.

## 5. Discussion

As one of the most diagnosed cancers, PCa remains a global health burden and challenges the current social healthcare system. In the past decade, significant improvement has been achieved in the field of disease etiology and treatment approaches. Considering the potentially large cases and dynamic changes of disease nature, more technological advances should be accomplished to better manage PCa. Right now, challenges lie in how to establishing a more precise system of risk classification based on clinical features of PCa. This is critical to distinguish those indolent cases, which don’t need to be heavily treated, from those that are potentially aggressive, which must be therapeutically intervened. Classification of disease subgroups based on computational histological pattern recognition and prediction of genomic features is now available for prostate cancer prognostication.

Other areas that deserve attention are the increasing number of treatment-induced NEPC, which presents in 10-17% of patients with CRPC [81], and repurpose of immunotherapy in PCa. As ADT and SG-ARIs are now the standard treatment for PCa, this number is expected to increase in the future. However, platinum-based chemotherapy is the only available treatment for NEPC [231], and inhibitors targeting AURKA or MYCN are still under investigation. PCa is a C-class tumor with low mutational burden and few T-cell infiltrations [232]. Most mutations are copy number alterations or gene structural rearrangements without the generation of neoantigen [233, 234]. This serves as a therapeutic barrier to immunotherapy, as a higher mutation burden and immune cell infiltration are associated with a better response rate [235, 236]. To repurpose immunotherapy for PCa, future research needs to clear this barrier, and this can be achieved through either combination therapy or identification of novel immune targets [237, 238].

Several new areas of PCa are being intensively studied, and their role in the disease onset and progression is still largely unknown. For example, senescence and senescence-associated secretory phenotype (SASP) are one of the potential mechanisms to promote cancer growth and metastasis [239]. Senescence and SASP are thought to secrete multiple immunosuppressive factors, which then recruit immunosuppressive cells to the site of the tumor. These immune cells then secrete cytokines and chemokines that lead to malignant phenotypes, preexistence of senescence, or immunosuppressive microenvironment [240-242]. This complex interaction between tumors in senescence and other factors is like a double-edged sword. On the one hand, tumors in a senescence state are dormant and thus stop growing, which is the major mechanism of tumor control by many chemotherapies [243, 244]. However, senescence is distinct from cell death, as tumors are still alive and possess secretory function, which is harmful for the long-term treatment and is responsible for disease relapse through the mechanisms mentioned above. How to navigate the dichotomous feature of senescence to the beneficial side should be considered in the future. Another investigation area is epigenetic therapy. Due to its slow onset and chronic progression, PCa is an excellent candidate for epigenetic therapy [245]. Multiple epigenetic changes, including DNA methylation and histone modifications, are involved in PCa initiation and progression, which are now considered hallmarks of human cancer [246]. There is already an existing epigenetic assay, named DOCUMENT, to help the early diagnosis of PCa [247]. However, whether a similar assay will guide the treatment needs to be verified. A complete and integrative epigenetic taxonomy of PCa under different conditions will help to solve this issue [248-250]. Admittedly, this review cannot cover everything in this field, and many new aspects of PCa are being uncovered. It is promising that in the near future, more therapeutic options will be available for PCa patients to better manage this disease and improve patients’ quality of life.

## References

[1] Siegel RL, Giaquinto AN, Jemal A: Cancer statistics, 2024. CA Cancer J Clin 2024. doi:10.3322/caac.21820 38230766

[2] Bechis SK, Carroll PR, Cooperberg MR: Impact of age at diagnosis on prostate cancer treatment and survival. Journal of clinical oncology: official journal of the American Society of Clinical Oncology 2011, 29(2):235–241. doi:10.1200/jco.2010.30.2075: found at the end of this article. 21135285 PMC3058279

[3] Gann PH: Risk factors for prostate cancer. Reviews in Urology 2002, 4 Suppl 5(Suppl 5): S3–s10.. PMC1476014 16986064

[4] Litwin MS, Tan HJ: The Diagnosis and Treatment of Prostate Cancer: A Review. Jama 2017, 317(24):2532–2542. doi:10.1001/jama.2017.7248 28655021

[5] Rebello RJ, Oing C, Knudsen KE, Loeb S, Johnson DC, Reiter RE, Gillessen S, Van der Kwast T, Bristow RG: Prostate cancer. Nature reviews Disease primers 2021, 7(1):9. doi:10.1038/s41572-020-00243-0 33542230

[6] Karantanos T, Corn PG, Thompson TC: Prostate cancer progression after androgen deprivation therapy: mechanisms of castrate resistance and novel therapeutic approaches. Oncogene 2013, 32(49):5501–5511. doi:10.1038/onc.2013.206. 23752182 PMC3908870

[7] Dellis A, Zagouri F, Liontos M, Mitropoulos D, Bamias A, Papatsoris AG: Management of advanced prostate cancer: A systematic review of existing guidelines and recommendations. Cancer treatment reviews 2019, 73:54–61. doi:10.1016/j.ctrv.2018.11.005 30623865

[8] Taylor BS, Schultz N, Hieronymus H, Gopalan A, Xiao Y, Carver BS, Arora VK, Kaushik P, Cerami E, Reva B : Integrative genomic profiling of human prostate cancer. Cancer Cell 2010, 18(1):11–22. doi:10.1016/j.ccr.2010.05.026. 20579941 PMC3198787

[9] Barbieri CE, Baca SC, Lawrence MS, Demichelis F, Blattner M, Theurillat JP, White TA, Stojanov P, Van Allen E, Stransky N : Exome sequencing identifies recurrent SPOP, FOXA1 and MED12 mutations in prostate cancer. Nature Genetics 2012, 44(6):685–689. doi:10.1038/ng.2279. 22610119 PMC3673022

[10] Cha HR, Lee JH, Ponnazhagan S: Revisiting Immunotherapy: A Focus on Prostate Cancer. Cancer research 2020, 80(8):1615–1623. doi:10.1158/0008-5472.Can-19-2948. 32066566 PMC7641094

[11] Bonaventura P, Shekarian T, Alcazer V, Valladeau-Guilemond J, Valsesia-Wittmann S, Amigorena S, Caux C, Depil S: Cold Tumors: A Therapeutic Challenge for Immunotherapy. Frontiers in immunology 2019, 10:168. doi:10.3389/fimmu.2019.00168. 30800125 PMC6376112

[12] Ittmann M: Anatomy and Histology of the Human and Murine Prostate. Cold Spring Harbor perspectives in medicine 2018, 8(5). doi:10.1101/cshperspect.a030346. PMC5932577 29038334

[13] Henry GH, Malewska A, Joseph DB, Malladi VS, Lee J, Torrealba J, Mauck RJ, Gahan JC, Raj GV, Roehrborn CG : A Cellular Anatomy of the Normal Adult Human Prostate and Prostatic Urethra. Cell reports 2018, 25(12):3530–3542.e3535. doi:10.1016/j.celrep.2018.11.086. 30566875 PMC6411034

[14] Chang JJ, Shinohara K, Bhargava V, Presti JC Jr., : Prospective evaluation of lateral biopsies of the peripheral zone for prostate cancer detection. The Journal of Urology 1998, 160(6 Pt 1):2111–2114. doi:10.1097/00005392-199812010-00044 9817334

[15] Wadhera P: An introduction to acinar pressures in BPH and prostate cancer. Nature Reviews Urology 2013, 10(6):358–366. doi:10.1038/nrurol.2013.86 23670181

[16] Franco OE, Jiang M, Strand DW, Peacock J, Fernandez S, Jackson RS 2nd, , Revelo MP, Bhowmick NA, Hayward SW: Altered TGF-β signaling in a subpopulation of human stromal cells promotes prostatic carcinogenesis. Cancer research 2011, 71(4):1272–1281. doi:10.1158/0008-5472.Can-10-3142. 21303979 PMC3076790

[17] Chua CW, Shibata M, Lei M, Toivanen R, Barlow LJ, Bergren SK, Badani KK, McKiernan JM, Benson MC, Hibshoosh H : Single luminal epithelial progenitors can generate prostate organoids in culture. Nature Cell Biology 2014, 16(10):951–961, 951–954. doi:10.1038/ncb3047. 25241035 PMC4183706

[18] Wang X, Kruithof-de Julio M, Economides KD, Walker D, Yu H, Halili MV, Hu YP, Price SM, Abate-Shen C, Shen MM: A luminal epithelial stem cell that is a cell of origin for prostate cancer. Nature 2009, 461(7263):495–500. doi:10.1038/nature08361. 19741607 PMC2800362

[19] Wang ZA, Toivanen R, Bergren SK, Chambon P, Shen MM: Luminal cells are favored as the cell of origin for prostate cancer. Cell reports 2014, 8(5):1339–1346. doi:10.1016/j.celrep.2014.08.002. 25176651 PMC4163115

[20] Goldstein AS, Huang J, Guo C, Garraway IP, Witte ON: Identification of a cell of origin for human prostate cancer. Science (New York, NY) 2010, 329(5991):568–571. doi:10.1126/science.1189992 PMC2917982 20671189

[21] Kwon OJ, Zhang L, Ittmann MM, Xin L: Prostatic inflammation enhances basal-to-luminal differentiation and accelerates initiation of prostate cancer with a basal cell origin. Proceedings of the National Academy of Sciences of the United States of America 2014, 111(5): E592–600. doi:10.1073/pnas.1318157111. 24367088 PMC3918789

[22] Stoyanova T, Cooper AR, Drake JM, Liu X, Armstrong AJ, Pienta KJ, Zhang H, Kohn DB, Huang J, Witte ON : Prostate cancer originating in basal cells progresses to adenocarcinoma propagated by luminal-like cells. Proceedings of the National Academy of Sciences of the United States of America 2013, 110(50):20111–20116. doi:10.1073/pnas.1320565110. 24282295 PMC3864278

[23] Wang ZA, Mitrofanova A, Bergren SK, Abate-Shen C, Cardiff RD, Califano A, Shen MM: Lineage analysis of basal epithelial cells reveals their unexpected plasticity and supports a cell-of-origin model for prostate cancer heterogeneity. Nature Cell Biology 2013, 15(3):274–283. doi:10.1038/ncb2697. 23434823 PMC3743266

[24] Choi N, Zhang B, Zhang L, Ittmann M, Xin L: Adult murine prostate basal and luminal cells are self-sustained lineages that can both serve as targets for prostate cancer initiation. Cancer Cell 2012, 21(2):253–265. doi:10.1016/j.ccr.2012.01.005. 22340597 PMC3285423

[25] De Marzo AM, Marchi VL, Epstein JI, Nelson WG: Proliferative inflammatory atrophy of the prostate: implications for prostatic carcinogenesis. The American journal of pathology 1999, 155(6):1985–1992. doi:10.1016/s0002-9440(10)65517-4. 10595928 PMC1866955

[26] Bostwick DG, Liu L, Brawer MK, Qian J: High-grade prostatic intraepithelial neoplasia. Reviews in Urology 2004, 6(4):171–179. . 16985598 PMC1472840

[27] Chughtai B, Forde JC, Thomas DD, Laor L, Hos-sack T, Woo HH, Te AE, Kaplan SA: Benign prostatic hyperplasia. Nature reviews Disease primers 2016, 2:16031. doi:10.1038/nrdp.2016.31 27147135

[28] Shoag JE, Nyame YA, Gulati R, Etzioni R, Hu JC: Reconsidering the Trade-offs of Prostate Cancer Screening. The New England journal of medicine 2020, 382(25):2465–2468. doi:10.1056/NEJMsb2000250. 32558473 PMC7491201

[29] Sturge J, Caley MP, Waxman J: Bone metastasis in prostate cancer: emerging therapeutic strategies. Nature reviews Clinical oncology 2011, 8(6):357–368. doi:10.1038/nrclinonc.2011.67 21556025

[30] Berish RB, Ali AN, Telmer PG, Ronald JA, Leong HS: Translational models of prostate cancer bone metastasis. Nature Reviews Urology 2018, 15(7):403–421. doi:10.1038/s41585-018-0020-2 29769644

[31] Gundem G, Van Loo P, Kremeyer B, Alexandrov LB, Tubio JMC, Papaemmanuil E, Brewer DS, Kallio HML, Högnäs G, Annala M : The evolutionary history of lethal metastatic prostate cancer. Nature 2015, 520(7547):353–357. doi:10.1038/nature14347. 25830880 PMC4413032

[32] Na TY, Schecterson L, Mendonsa AM, Gumbiner BM: The functional activity of E-cadherin controls tumor cell metastasis at multiple steps. Proceedings of the National Academy of Sciences of the United States of America 2020, 117(11):5931–5937. doi:10.1073/pnas.1918167117. 32127478 PMC7084067

[33] Torrano V, Valcarcel-Jimenez L, Cortazar AR, Liu X, Urosevic J, Castillo-Martin M, Fernández-Ruiz S, Morciano G, Caro-Maldonado A, Guiu M : The metabolic co-regulator PGC1α suppresses prostate cancer metastasis. Nature Cell Biology 2016, 18(6):645–656. doi:10.1038/ncb3357. 27214280 PMC4884178

[34] Stultz J, Fong L: How to turn up the heat on the cold immune microenvironment of metastatic prostate cancer. Prostate cancer and prostatic diseases 2021, 24(3):697–717. doi:10.1038/s41391-021-00340-5. 33820953 PMC8384622

[35] Gartrell BA, Saad F: Managing bone metastases and reducing skeletal-related events in prostate cancer. Nature reviews Clinical oncology 2014, 11(6):335–345. doi:10.1038/nrclinonc.2014.70 24821212

[36] Crawford ED, Stone NN, Yu EY, Koo PJ, Freed-land SJ, Slovin SF, Gomella LG, Berger ER, Keane TE, Sieber P : Challenges and recommendations for early identification of metastatic disease in prostate cancer. Urology 2014, 83(3):664–669. doi:10.1016/j.urology.2013.10.026 24411213

[37] The Molecular Taxonomy of Primary Prostate Cancer. Cell 2015, 163(4):1011–1025. doi:10.1016/j.cell.2015.10.025. 26544944 PMC4695400

[38] Robinson D, Van Allen EM, Wu YM, Schultz N, Lonigro RJ, Mosquera JM, Montgomery B, Taplin ME, Pritchard CC, Attard G : Integrative clinical genomics of advanced prostate cancer. Cell 2015, 161(5):1215–1228. doi:10.1016/j.cell.2015.05.001. 26000489 PMC4484602

[39] Fraser M, Sabelnykova VY, Yamaguchi TN, Heisler LE, Livingstone J, Huang V, Shiah YJ, Yousif F, Lin X, Masella AP : Genomic hallmarks of localized, non-indolent prostate cancer. Nature 2017, 541(7637):359–364. doi:10.1038/nature20788 28068672

[40] Clark JP, Cooper CS: ETS gene fusions in prostate cancer. Nature Reviews Urology 2009, 6(8):429–439. doi:10.1038/nrurol.2009.127 19657377

[41] Feng FY, Brenner JC, Hussain M, Chinnai-yan AM: Molecular pathways: targeting ETS gene fusions in cancer. Clinical cancer research: an official journal of the American Association for Cancer Research 2014, 20(17):4442–4448. doi:10.1158/1078-0432.Ccr-13-0275. 24958807 PMC4155001

[42] Li J, Xu C, Lee HJ, Ren S, Zi X, Zhang Z, Wang H, Yu Y, Yang C, Gao X: A genomic and epigenomic atlas of prostate cancer in Asian populations. Nature 2020, 580(7801):93–99. 32238934 10.1038/s41586-020-2135-x

[43] Theurillat JP, Udeshi ND, Errington WJ, Svinkina T, Baca SC, Pop M, Wild PJ, Blattner M, Groner AC, Rubin MA : Prostate cancer. Ubiquitylome analysis identifies dysregulation of effector substrates in SPOP-mutant prostate cancer. Science (New York, NY) 2014, 346(6205):85–89. doi:10.1126/science.1250255. PMC4257137 25278611

[44] Blattner M, Liu D, Robinson BD, Huang D, Poliakov A, Gao D, Nataraj S, Deonarine LD, Augello MA, Sailer V : SPOP Mutation Drives Prostate Tumorigenesis In Vivo through Coordinate Regulation of PI3K/mTOR and AR Signaling. Cancer cell 2017, 31(3):436–451. doi:10.1016/j.ccell.2017.02.004. 28292441 PMC5384998

[45] Dai X, Gan W, Li X, Wang S, Zhang W, Huang L, Liu S, Zhong Q, Guo J, Zhang J : Prostate cancer-associated SPOP mutations confer resistance to BET inhibitors through stabilization of BRD4. Nat Med 2017, 23(9):1063–1071. doi:10.1038/nm.4378. 28805820 PMC5625299

[46] Parolia A, Cieslik M, Chu SC, Xiao L, Ouchi T, Zhang Y, Wang X, Vats P, Cao X, Pitchiaya S : Distinct structural classes of activating FOXA1 alterations in advanced prostate cancer. Nature 2019, 571(7765):413–418. doi:10.1038/s41586-019-1347-4. 31243372 PMC6661908

[47] Adams EJ, Karthaus WR, Hoover E, Liu D, Gruet A, Zhang Z, Cho H, DiLoreto R, Chhangawala S, Liu Y : FOXA1 mutations alter pioneering activity, differentiation, and prostate cancer phenotypes. Nature 2019, 571(7765):408–412. doi:10.1038/s41586-019-1318-9. 31243370 PMC6661172

[48] An J, Ren S, Murphy SJ, Dalangood S, Chang C, Pang X, Cui Y, Wang L, Pan Y, Zhang X : Truncated ERG Oncoproteins from TMPRSS2-ERG Fusions Are Resistant to SPOP-Mediated Proteasome Degradation. Molecular Cell 2015, 59(6):904–916. doi:10.1016/j.molcel.2015.07.025 26344096

[49] Pearson HB, Li J, Meniel VS, Fennell CM, Waring P, Montgomery KG, Rebello RJ, Macpherson AA, Koushyar S, Furic L : Identification of Pik-3ca Mutation as a Genetic Driver of Prostate Cancer That Cooperates with Pten Loss to Accelerate Progression and Castration-Resistant Growth. Cancer discovery 2018, 8(6):764–779. doi:10.1158/2159-8290.Cd-17-0867 29581176

[50] Herberts C, Murtha AJ, Fu S, Wang G, Schönlau E, Xue H, Lin D, Gleave A, Yip S, Angeles A : Activating AKT1 and PIK3CA Mutations in Metastatic Castration-Resistant Prostate Cancer. European Urology 2020, 78(6):834–844. doi:10.1016/j.eururo.2020.04.058 32451180

[51] Jamaspishvili T, Berman DM, Ross AE, Scher HI, De Marzo AM, Squire JA, Lotan TL: Clinical implications of PTEN loss in prostate cancer. Nature Reviews Urology 2018, 15(4):222–234. doi:10.1038/nrurol.2018.9. 29460925 PMC7472658

[52] Kypta RM, Waxman J: Wnt/β-catenin signalling in prostate cancer. Nature Reviews Urology 2012, 9(8):418–428. doi:10.1038/nrurol.2012.116 22710668

[53] Grasso CS, Wu YM, Robinson DR, Cao X, Dhanasekaran SM, Khan AP, Quist MJ, Jing X, Lonigro RJ, Brenner JC : The mutational landscape of lethal castration-resistant prostate cancer. Nature 2012, 487(7406):239–243. doi:10.1038/nature11125. 22722839 PMC3396711

[54] Murillo-Garzón V, Kypta R: WNT signalling in prostate cancer. Nature Reviews Urology 2017, 14(11):683–696. doi:10.1038/nrurol.2017.144 28895566

[55] Miyamoto DT, Zheng Y, Wittner BS, Lee RJ, Zhu H, Broderick KT, Desai R, Fox DB, Brannigan BW, Trautwein J : RNA-Seq of single prostate CTCs implicates noncanonical Wnt signaling in antiandrogen resistance. Science 2015, 349(6254):1351–1356. doi:10.1126/science.aab0917. 26383955 PMC4872391

[56] Sheng X, Nenseth HZ, Qu S, Kuzu OF, Frahnow T, Simon L, Greene S, Zeng Q, Fazli L, Rennie PS : IRE1α-XBP1s pathway promotes prostate cancer by activating c-MYC signaling. Nature Communications 2019, 10(1):323. doi:10.1038/s41467-018-08152-3: remaining authors declare no competing interests. PMC6345973 30679434

[57] Koh CM, Bieberich CJ, Dang CV, Nelson WG, Yegnasubramanian S, De Marzo AM: MYC and Prostate Cancer. Genes & cancer 2010, 1(6):617–628. doi:10.1177/1947601910379132. 21779461 PMC3092219

[58] Hubbard GK, Mutton LN, Khalili M, McMullin RP, Hicks JL, Bianchi-Frias D, Horn LA, Kulac I, Moubarek MS, Nelson PS : Combined MYC Activation and Pten Loss Are Sufficient to Create Genomic Instability and Lethal Metastatic Prostate Cancer. Cancer research 2016, 76(2):283–292. doi:10.1158/0008-5472.Can-14-3280. 26554830 PMC5006678

[59] Robinson JL, Hickey TE, Warren AY, Vowler SL, Carroll T, Lamb AD, Papoutsoglou N, Neal DE, Tilley WD, Carroll JS: Elevated levels of FOXA1 facilitate androgen receptor chromatin binding resulting in a CRPC-like phenotype. Oncogene 2014, 33(50):5666–5674. 24292680 10.1038/onc.2013.508 PMC4051595

[60] Hsieh CL, Botta G, Gao S, Li T, Van Allen EM, Treacy DJ, Cai C, He HH, Sweeney CJ, Brown M : PLZF, a tumor suppressor genetically lost in metastatic castration-resistant prostate cancer, is a mediator of resistance to androgen deprivation therapy. Cancer research 2015, 75(10):1944–1948. doi:10.1158/0008-5472.Can-14-3602. 25808865 PMC4433564

[61] Hodgson MC, Astapova I, Cheng S, Lee LJ, Verhoeven MC, Choi E, Balk SP, Hollenberg AN: The androgen receptor recruits nuclear receptor CoRepressor (N-CoR) in the presence of mifepristone via its N and C termini, revealing a novel molecular mechanism for androgen receptor antagonists. The Journal of Biological Chemistry 2005, 280(8):6511–6519. doi:10.1074/jbc.M408972200 15598662

[62] Stopsack KH, Nandakumar S, Wibmer AG, Haywood S, Weg ES, Barnett ES, Kim CJ, Carbone EA, Vasselman SE, Nguyen B : Oncogenic Genomic Alterations, Clinical Phenotypes, and Outcomes in Metastatic Castration-Sensitive Prostate Cancer. Clinical cancer research: an official journal of the American Association for Cancer Research 2020, 26(13):3230–3238. doi:10.1158/1078-0432.Ccr-20-0168. 32220891 PMC7334067

[63] Korpal M, Korn JM, Gao X, Rakiec DP, Ruddy DA, Doshi S, Yuan J, Kovats SG, Kim S, Cooke VG : An F876L mutation in androgen receptor confers genetic and phenotypic resistance to MDV3100 (enzalutamide). Cancer discovery 2013, 3(9):1030–1043. doi:10.1158/2159-8290.Cd-13-0142 23842682

[64] Joseph JD, Lu N, Qian J, Sensintaffar J, Shao G, Brigham D, Moon M, Maneval EC, Chen I, Darimont B : A clinically relevant androgen receptor mutation confers resistance to second-generation antiandrogens enzalutamide and ARN-509. Cancer Discov 2013, 3(9):1020–1029. doi:10.1158/2159-8290.CD-13-0226 23779130

[65] Watson PA, Arora VK, Sawyers CL: Emerging mechanisms of resistance to androgen receptor inhibitors in prostate cancer. Nat Rev Cancer 2015, 15(12):701–711. doi:10.1038/nrc4016. 26563462 PMC4771416

[66] Dehm SM, Schmidt LJ, Heemers HV, Vessella RL, Tindall DJ: Splicing of a novel androgen receptor exon generates a constitutively active androgen receptor that mediates prostate cancer therapy resistance. Cancer research 2008, 68(13):5469–5477. doi:10.1158/0008-5472.Can-08-0594. 18593950 PMC2663383

[67] Hu R, Dunn TA, Wei S, Isharwal S, Veltri RW, Humphreys E, Han M, Partin AW, Vessella RL, Isaacs WB : Ligand-independent androgen receptor variants derived from splicing of cryptic exons signify hormone-refractory prostate cancer. Cancer Res 2009, 69(1):16–22. doi:10.1158/0008-5472.CAN-08-2764. 19117982 PMC2614301

[68] Guo Z, Yang X, Sun F, Jiang R, Linn DE, Chen H, Chen H, Kong X, Melamed J, Tepper CG : A novel androgen receptor splice variant is up-regulated during prostate cancer progression and promotes androgen depletion-resistant growth. Cancer research 2009, 69(6):2305–2313. doi:10.1158/0008-5472.Can-08-3795. 19244107 PMC2672822

[69] Paschalis A, Sharp A, Welti JC, Neeb A, Raj GV, Luo J, Plymate SR, de Bono JS: Alternative splicing in prostate cancer. Nature reviews Clinical oncology 2018, 15(11):663–675. doi:10.1038/s41571-018-0085-0 30135575

[70] Pritchard CC, Mateo J, Walsh MF, De Sarkar N, Abida W, Beltran H, Garofalo A, Gulati R, Carreira S, Eeles R : Inherited DNA-Repair Gene Mutations in Men with Metastatic Prostate Cancer. The New England journal of medicine 2016, 375(5):443–453. doi:10.1056/NEJ-Moa1603144. 27433846 PMC4986616

[71] Mateo J, Boysen G, Barbieri CE, Bryant HE, Castro E, Nelson PS, Olmos D, Pritchard CC, Rubin MA, de Bono JS: DNA Repair in Prostate Cancer: Biology and Clinical Implications. European Urology 2017, 71(3):417–425. doi:10.1016/j.eururo.2016.08.037 27590317

[72] Abida W, Cheng ML, Armenia J, Middha S, Autio KA, Vargas HA, Rathkopf D, Morris MJ, Danila DC, Slovin SF : Analysis of the Prevalence of Microsatellite Instability in Prostate Cancer and Response to Immune Checkpoint Blockade. JAMA Oncol 2019, 5(4):471–478. doi:10.1001/jamaoncol.2018.5801. 30589920 PMC6459218

[73] Polkinghorn WR, Parker JS, Lee MX, Kass EM, Spratt DE, Iaquinta PJ, Arora VK, Yen WF, Cai L, Zheng D : Androgen receptor signaling regulates DNA repair in prostate cancers. Cancer discovery 2013, 3(11):1245–1253. doi:10.1158/2159-8290.Cd-13-0172. 24027196 PMC3888815

[74] O’Neil NJ, Bailey ML, Hieter P: Synthetic lethality and cancer. Nature Reviews Genetics 2017, 18(10):613–623. doi:10.1038/nrg.2017.47 28649135

[75] Lord CJ, Ashworth A: PARP inhibitors: Synthetic lethality in the clinic. Science (New York, NY) 2017, 355(6330):1152–1158. doi:10.1126/science.aam7344. PMC6175050 28302823

[76] Beltran H, Rickman DS, Park K, Chae SS, Sboner A, MacDonald TY, Wang Y, Sheikh KL, Terry S, Tagawa ST : Molecular characterization of neuroendocrine prostate cancer and identification of new drug targets. Cancer Discovery 2011, 1(6):487–495. doi:10.1158/2159-8290.Cd-11-0130. 22389870 PMC3290518

[77] Beltran H, Prandi D, Mosquera JM, Benelli M, Puca L, Cyrta J, Marotz C, Giannopoulou E, Chakravarthi BV, Varambally S : Divergent clonal evolution of castration-resistant neuroendocrine prostate cancer. Nat Med 2016, 22(3):298–305. doi:10.1038/nm.4045. 26855148 PMC4777652

[78] Ku SY, Rosario S, Wang Y, Mu P, Seshadri M, Goodrich ZW, Goodrich MM, Labbé DP, Gomez EC, Wang J : Rb1 and Trp53 cooperate to suppress prostate cancer lineage plasticity, metastasis, and antiandrogen resistance. Science (New York, NY) 2017, 355(6320):78–83. doi:10.1126/science.aah4199. PMC5367887 28059767

[79] Park JW, Lee JK, Sheu KM, Wang L, Balanis NG, Nguyen K, Smith BA, Cheng C, Tsai BL, Cheng D : Reprogramming normal human epithelial tissues to a common, lethal neuroendocrine cancer lineage. Science (New York, NY) 2018, 362(6410):91–95. doi:10.1126/science.aat5749. PMC6414229 30287662

[80] Nyquist MD, Corella A, Coleman I, De Sarkar N, Kaipainen A, Ha G, Gulati R, Ang L, Chatterjee P, Lucas J : Combined TP53 and RB1 Loss Promotes Prostate Cancer Resistance to a Spectrum of Therapeutics and Confers Vulnerability to Replication Stress. Cell Reports 2020, 31(8):107669. doi:10.1016/j.celrep.2020.107669. 32460015 PMC7453577

[81] Wang Y, Wang Y, Ci X, Choi SYC, Crea F, Lin D, Wang Y: Molecular events in neuroendocrine prostate cancer development. Nature Reviews Urology 2021, 18(10):581–596. doi:10.1038/s41585-021-00490-0 34290447 PMC10802813

[82] Zou M, Toivanen R, Mitrofanova A, Floch N, Hayati S, Sun Y, Le Magnen C, Chester D, Mostaghel EA, Califano A : Transdifferentiation as a Mechanism of Treatment Resistance in a Mouse Model of Castration-Resistant Prostate Cancer. Cancer Discovery 2017, 7(7):736–749. doi:10.1158/2159-8290.Cd-16-1174. 28411207 PMC5501744

[83] Guo H, Ci X, Ahmed M, Hua JT, Soares F, Lin D, Puca L, Vosoughi A, Xue H, Li E : ONECUT2 is a driver of neuroendocrine prostate cancer. Nat Commun 2019, 10(1):278. doi:10.1038/s41467-018-08133-6. 30655535 PMC6336817

[84] Davies A, Nouruzi S, Ganguli D, Namekawa T, Thaper D, Linder S, Karaoğlanoğlu F, Omur ME, Kim S, Kobelev M : An androgen receptor switch underlies lineage infidelity in treatment-resistant prostate cancer. Nature Cell Biology 2021, 23(9):1023–1034. doi:10.1038/s41556-021-00743-5. 34489572 PMC9012003

[85] De Marzo AM, Platz EA, Sutcliffe S, Xu J, Gronberg H, Drake CG, Nakai Y, Isaacs WB, Nelson WG: Inflammation in prostate carcinogenesis. Nat Rev Cancer 2007, 7(4):256–269. doi:10.1038/nrc2090. 17384581 PMC3552388

[86] Sfanos KS, De Marzo AM: Prostate cancer and inflammation: the evidence. Histopathology 2012, 60(1):199–215. doi:10.1111/j.1365-2559.2011.04033.x. 22212087 PMC4029103

[87] de Bono JS, Guo C, Gurel B, De Marzo AM, Sfanos KS, Mani RS, Gil J, Drake CG, Alimonti A: Prostate carcinogenesis: inflammatory storms. Nature Reviews Cancer 2020, 20(8):455–469. doi:10.1038/s41568-020-0267-9 32546840

[88] Sfanos KS, Yegnasubramanian S, Nelson WG, De Marzo AM: The inflammatory microenvironment and microbiome in prostate cancer development. Nature Reviews Urology 2018, 15(1):11–24. doi:10.1038/nrurol.2017.167 29089606

[89] Sfanos KS, Canene-Adams K, Hempel H, Yu SH, Simons BW, Schaeffer AJ, Schaeffer EM, Nelson WG, DeMarzo AM: Bacterial Prostatitis Enhanc-es 2-Amino-1-Methyl-6-Phenylimidazo[4,5-b] Pyridine (PhIP)-Induced Cancer at Multiple Sites. Cancer prevention research (Philadelphia, Pa) 2015, 8(8):683–692. doi:10.1158/1940-6207.Capr-15-0090. 25990088 PMC4527940

[90] Kakegawa T, Bae Y, Ito T, Uchida K, Sekine M, Nakajima Y, Furukawa A, Suzuki Y, Kumagai J, Akashi T : Frequency of Propionibac-terium acnes Infection in Prostate Glands with Negative Biopsy Results Is an Independent Risk Factor for Prostate Cancer in Patients with Increased Serum PSA Titers. PloS one 2017, 12(1):e0169984. doi:10.1371/journal.pone.0169984. 28081259 PMC5231393

[91] Parent ME, Désy M, Siemiatycki J: Does exposure to agricultural chemicals increase the risk of prostate cancer among farmers? McGill Journal of Medicine: MJM: an international forum for the advancement of medical sciences by students 2009, 12(1):70–77. . 19753293 PMC2687920

[92] Krstev S, Knutsson A: Occupational Risk Factors for Prostate Cancer: A Meta-analysis. Journal of Cancer Prevention 2019, 24(2):91–111. doi:10.15430/jcp.2019.24.2.91. 31360689 PMC6619854

[93] Barrington WE, Schenk JM, Etzioni R, Arnold KB, Neuhouser ML, Thompson IM Jr., Lucia MS, Kristal AR: Difference in Association of Obesity With Prostate Cancer Risk Between US African American and Non-Hispanic White Men in the Selenium and Vitamin E Cancer Prevention Trial (SELECT). JAMA Oncology 2015, 1(3):342–349. doi:10.1001/jamaoncol.2015.0513. 26181184 PMC4570268

[94] Wiseman H, Halliwell B: Damage to DNA by reactive oxygen and nitrogen species: role in inflammatory disease and progression to cancer. The Biochemical journal 1996, 313 ( Pt 1)(Pt 1):17–29. doi:10.1042/bj3130017. 8546679 PMC1216878

[95] Coussens LM, Werb Z: Inflammation and cancer. Nature 2002, 420(6917):860–867. doi:10.1038/nature01322. 12490959 PMC2803035

[96] Greten FR, Grivennikov SI: Inflammation and Cancer: Triggers, Mechanisms, and Consequences. Immunity 2019, 51(1):27–41. doi:10.1016/j.immuni.2019.06.025. 31315034 PMC6831096

[97] Le Magnen C, Virk RK, Dutta A, Kim JY, Pan-ja S, Lopez-Bujanda ZA, Califano A, Drake CG, Mitrofanova A, Abate-Shen C: Cooperation of loss of NKX3.1 and inflammation in prostate cancer initiation. Disease models & mechanisms 2018, 11(11). doi:10.1242/dmm.035139. PMC6262819 30266798

[98] Song H, Zhang B, Watson MA, Humphrey PA, Lim H, Milbrandt J: Loss of Nkx3.1 leads to the activation of discrete downstream target genes during prostate tumorigenesis. Oncogene 2009, 28(37):3307–3319. doi:10.1038/onc.2009.181. 19597465 PMC2746257

[99] Bethel CR, Faith D, Li X, Guan B, Hicks JL, Lan F, Jenkins RB, Bieberich CJ, De Marzo AM: Decreased NKX3.1 protein expression in focal prostatic atrophy, prostatic intraepithelial neoplasia, and adenocarcinoma: association with Gleason score and chromosome 8p deletion. Cancer research 2006, 66(22):10683–10690. doi:10.1158/0008-5472.Can-06-0963 17108105

[100] Maxwell PJ, Coulter J, Walker SM, McKechnie M, Neisen J, McCabe N, Kennedy RD, Salto-Tellez M, Albanese C, Waugh DJ: Potentiation of inflammatory CXCL8 signalling sustains cell survival in PTEN-deficient prostate carcinoma. European Urology 2013, 64(2):177–188. doi:10.1016/j.eururo.2012.08.032. 22939387 PMC4185293

[101] Packer JR, Maitland NJ: The molecular and cellular origin of human prostate cancer. Biochimica et biophysica acta 2016, 1863(6 Pt A):1238–1260. doi:10.1016/j.bbam-cr.2016.02.016 26921821

[102] Ashok A, Keener R, Rubenstein M, Stookey S, Bajpai S, Hicks J, Alme AK, Drake CG, Zheng Q, Trabzonlu L : Consequences of interleukin 1β-triggered chronic inflammation in the mouse prostate gland: Altered architecture associated with prolonged CD4(+) infiltration mimics human proliferative inflammatory atrophy. The Prostate 2019, 79(7):732–745. doi:10.1002/pros.23784 30900284

[103] Rojas A, Liu G, Coleman I, Nelson PS, Zhang M, Dash R, Fisher PB, Plymate SR, Wu JD: IL-6 promotes prostate tumorigenesis and progression through autocrine cross-activation of IGF-IR. Oncogene 2011, 30(20):2345–2355. doi:10.1038/onc.2010.605. 21258401 PMC3112005

[104] Lopez-Bujanda ZA, Haffner MC, Chaimowitz MG, Chowdhury N, Venturini NJ, Patel RA, Obradovic A, Hansen CS, Jacków J, Maynard JP : Castration-mediated IL-8 promotes myeloid infiltration and prostate cancer progression. Nature Cancer 2021, 2(8):803–818. doi:10.1038/s43018-021-00227-3 35122025 PMC9169571

[105] Malinen M, Niskanen EA, Kaikkonen MU, Palvimo JJ: Crosstalk between androgen and pro-inflammatory signaling remodels androgen receptor and NF-κB cistrome to reprogram the prostate cancer cell transcriptome. Nucleic acids research 2017, 45(2):619–630. doi:10.1093/nar/gkw855. 27672034 PMC5314794

[106] Banzola I, Mengus C, Wyler S, Hudolin T, Manzella G, Chiarugi A, Boldorini R, Sais G, Schmidli TS, Chiffi G : Expression of Indoleamine 2,3-Dioxygenase Induced by IFN-γ and TNF-α as Potential Biomarker of Prostate Cancer Progression. Frontiers in immunology 2018, 9:1051. doi:10.3389/fimmu.2018.01051. 29896191 PMC5986916

[107] Calcinotto A, Spataro C, Zagato E, Di Mitri D, Gil V, Crespo M, De Bernardis G, Losa M, Mirenda M, Pasquini E : IL-23 secreted by myeloid cells drives castration-resistant prostate cancer. Nature 2018, 559(7714):363–369. doi:10.1038/s41586-018-0266-0. 29950727 PMC6461206

[108] Liu C, Zhu Y, Lou W, Cui Y, Evans CP, Gao AC: Inhibition of constitutively active Stat3 reverses enzalutamide resistance in LN-CaP derivative prostate cancer cells. Prostate 2014, 74(2):201–209. doi:10.1002/pros.22741. 24307657 PMC4437226

[109] Gupta S, Pungsrinont T, Ženata O, Neubert L, Vrzal R, Baniahmad A: Interleukin-23 Represses the Level of Cell Senescence Induced by the Androgen Receptor Antagonists Enzalut-amide and Darolutamide in Castration-Resistant Prostate Cancer Cells. Hormones & cancer 2020, 11(3-4):182–190. doi:10.1007/s12672-020-00391-5. 32562083 PMC7335377

[110] Mottet N, Bellmunt J, Bolla M, Briers E, Cumberbatch MG, De Santis M, Fossati N, Gross T, Henry AM, Joniau S : EAU-ESTRO-SIOG Guidelines on Prostate Cancer. Part 1: Screening, Diagnosis, and Local Treatment with Curative Intent. European Urology 2017, 71(4):618–629. doi:10.1016/j.eururo.2016.08.003 27568654

[111] Lotan TL, Epstein JI: Clinical implications of changing definitions within the Gleason grading system. Nature Reviews Urology 2010, 7(3):136–142. doi:10.1038/nrurol.2010.9 20157302

[112] Epstein JI: Prostate cancer grading: a decade after the 2005 modified system. Modern pathology: an official journal of the United States and Canadian Academy of Pathology, Inc 2018, 31(S1): S47–63. doi:10.1038/modpathol.2017.133 29297487

[113] Epstein JI, Egevad L, Amin MB, Delahunt B, Srigley JR, Humphrey PA: The 2014 International Society of Urological Pathology (ISUP) Consensus Conference on Gleason Grading of Prostatic Carcinoma: Definition of Grading Patterns and Proposal for a New Grading System. The American journal of surgical pathology 2016, 40(2):244–252. doi:10.1097/pas.0000000000000530 26492179

[114] Stark JR, Perner S, Stampfer MJ, Sinnott JA, Finn S, Eisenstein AS, Ma J, Fiorentino M, Kurth T, Loda M : Gleason score and lethal prostate cancer: does 3 + 4 = 4 + 3? Journal of clinical oncology: official journal of the American Society of Clinical Oncology 2009, 27(21):3459–3464. doi:10.1200/jco.2008.20.4669: found at the end of this article. 19433685 PMC2717753

[115] Wright JL, Salinas CA, Lin DW, Kolb S, Koopmeiners J, Feng Z, Stanford JL: Prostate cancer-specific mortality and Gleason 7 disease differences in prostate cancer outcomes between cases with Gleason 4 + 3 and Gleason 3 + 4 tumors in a population-based cohort. The Journal of Urology 2009, 182(6):2702–2707. doi:10.1016/j.juro.2009.08.026. 19836772 PMC2828768

[116] Welch HG, Albertsen PC: Prostate cancer diagnosis and treatment after the introduction of prostate-specific antigen screening: 1986-2005. Journal of the National Cancer Institute 2009, 101(19):1325–1329. doi:10.1093/jnci/djp278. 19720969 PMC2758309

[117] Lilja H, Ulmert D, Vickers AJ: Prostate-specific antigen and prostate cancer: prediction, detection and monitoring. Nature Reviews Cancer 2008, 8(4):268–278. doi:10.1038/nrc2351 18337732

[118] D’Amico AV, Renshaw AA, Sussman B, Chen MH: Pretreatment PSA velocity and risk of death from prostate cancer following external beam radiation therapy. Jama 2005, 294(4):440–447. doi:10.1001/jama.294.4.440 16046650

[119] Zagars GK, Pollack A, Kavadi VS, von Es-chenbach AC: Prostate-specific antigen and radiation therapy for clinically localized prostate cancer. International journal of radiation oncology, biology, physics 1995, 32(2):293–306. doi:10.1016/0360-3016(95)00077-c 7538498

[120] Kwak C, Jeong SJ, Park MS, Lee E, Lee SE: Prognostic significance of the nadir prostate-specific antigen level after hormone therapy for prostate cancer. The Journal of Urology 2002, 168(3):995–1000. doi:10.1097/01.ju.0000024925.67014.21 12187207

[121] Geara FB, Bulbul M, Khauli RB, Andraos TY, Abboud M, Al Mousa A, Sarhan N, Salem A, Ghatasheh H, Alnsour A : Nadir PSA is a strong predictor of treatment outcome in intermediate and high risk localized prostate cancer patients treated by definitive external beam radiotherapy and androgen deprivation. Radiation oncology (London, England) 2017, 12(1):149. doi:10.1186/s13014-017-0884-y. 28882187 PMC5590195

[122] Partin AW, Pound CR, Clemens JQ, Epstein JI, Walsh PC: Serum PSA after anatomic radical prostatectomy. The Johns Hopkins experience after 10 years. The Urologic Clinics of North America 1993, 20(4):713–725. 7505980

[123] Pound CR, Partin AW, Eisenberger MA, Chan DW, Pearson JD, Walsh PC: Natural history of progression after PSA elevation following radical prostatectomy. Jama 1999, 281(17):1591–1597. doi:10.1001/jama.281.17.1591 10235151

[124] Stephenson AJ, Kattan MW, Eastham JA, Dotan ZA, Bianco FJ Jr., Lilja H, Scardino PT: Defining biochemical recurrence of prostate cancer after radical prostatectomy: a proposal for a standardized definition. Journal of clinical oncology: official journal of the American Society of Clinical Oncology 2006, 24(24):3973–3978. doi:10.1200/jco.2005.04.0756 16921049

[125] Wojno KJ, Costa FJ, Cornell RJ, Small JD, Pasin E, Van Criekinge W, Bigley JW, Van Neste L: Reduced rate of repeated prostate biopsies observed in ConfirmMDx clinical utility field study. American health & drug benefits 2014, 7(3):129. 24991397 PMC4070628

[126] Serefoglu EC, Altinova S, Ugras NS, Akin-cioglu E, Asil E, Balbay MD: How reliable is the 12-core prostate biopsy procedure in the detection of prostate cancer? Canadian Urological Association journal = Journal de l’Association des urologues du Canada 2013, 7(5–6):E293–298. doi:10.5489/cuaj.11224. PMC3668408 22398204

[127] Bjurlin MA, Carter HB, Schellhammer P, Cookson MS, Gomella LG, Troyer D, Wheeler TM, Schlossberg S, Penson DF, Taneja SS: Optimization of initial prostate biopsy in clinical practice: sampling, labeling, and specimen processing. The Journal of Urology 2013, 189(6):2039–2046. doi:10.1016/j.juro.2013.02.072. 23485507 PMC3925148

[128] Etzioni R, Penson DF, Legler JM, di Tommaso D, Boer R, Gann PH, Feuer EJ: Overdiagnosis due to prostate-specific antigen screening: lessons from U.S. prostate cancer incidence trends. Journal of the National Cancer Institute 2002, 94(13):981–990. doi:10.1093/jnci/94.13.981 12096083

[129] Loeb S, Bjurlin MA, Nicholson J, Tammela TL, Penson DF, Carter HB, Carroll P, Etzioni R: Overdiagnosis and overtreatment of prostate cancer. European Urology 2014, 65(6):1046–1055. doi:10.1016/j.eururo.2013.12.062. 24439788 PMC4113338

[130] Welch HG, Black WC: Overdiagnosis in cancer. Journal of the National Cancer Institute 2010, 102(9):605–613. doi:10.1093/jnci/djq099 20413742

[131] Draisma G, Boer R, Otto SJ, van der Cruijsen IW, Damhuis RA, Schröder FH, de Koning HJ: Lead times and overdetection due to prostate-specific antigen screening: estimates from the European Randomized Study of Screening for Prostate Cancer. Journal of the National Cancer Institute 2003, 95(12):868–878. doi:10.1093/jnci/95.12.868 12813170

[132] Draisma G, Etzioni R, Tsodikov A, Mariotto A, Wever E, Gulati R, Feuer E, de Koning H: Lead time and overdiagnosis in prostate-specific antigen screening: importance of methods and context. Journal of the National Cancer Institute 2009, 101(6):374–383. doi:10.1093/jnci/djp001. 19276453 PMC2720697

[133] Carter HB: Prostate cancers in men with low PSA levels--must we find them? The New England journal of medicine 2004, 350(22):2292–2294. doi:10.1056/NEJMe048003. 15163780 PMC3474980

[134] Bluemn EG, Coleman IM, Lucas JM, Coleman RT, Hernandez-Lopez S, Tharakan R, Bianchi-Frias D, Dumpit RF, Kaipainen A, Corella AN : Androgen Receptor Pathway-Independent Prostate Cancer Is Sustained through FGF Signaling. Cancer cell 2017, 32(4):474–489.e476. doi:10.1016/j.ccell.2017.09.003. 29017058 PMC5750052

[135] Weinreb JC, Barentsz JO, Choyke PL, Cornud F, Haider MA, Macura KJ, Margolis D, Schnall MD, Shtern F, Tempany CM : PI-RADS Prostate Imaging - Reporting and Data System: 2015, Version 2. European urology 2016, 69(1):16–40. doi:10.1016/j.eururo.2015.08.052. 26427566 PMC6467207

[136] Kasivisvanathan V, Rannikko AS, Borghi M, Panebianco V, Mynderse LA, Vaarala MH, Briganti A, Budäus L, Hellawell G, Hindley RG : MRI-Targeted or Standard Biopsy for Prostate-Cancer Diagnosis. The New England journal of medicine 2018, 378(19):1767–1777. doi:10.1056/NEJMoa1801993 29552975 PMC9084630

[137] Klotz L, Chin J, Black PC, Finelli A, Anidjar M, Bladou F, Mercado A, Levental M, Ghai S, Chang SD : Comparison of Multiparametric Magnetic Resonance Imaging-Targeted Biopsy With Systematic Transrectal Ultrasonography Biopsy for Biopsy-Naive Men at Risk for Prostate Cancer: A Phase 3 Randomized Clinical Trial. JAMA Oncol 2021, 7(4):534–542. doi:10.1001/jamaoncol.2020.7589. 33538782 PMC7863017

[138] Albertsen PC, Moore DF, Shih W, Lin Y, Li H, Lu-Yao GL: Impact of comorbidity on survival among men with localized prostate cancer. Journal of clinical oncology: official journal of the American Society of Clinical Oncology 2011, 29(10):1335–1341. doi:10.1200/jco.2010.31.2330: found at the end of this article. 21357791 PMC3084001

[139] Schröder FH, Hugosson J, Roobol MJ, Tammela TL, Ciatto S, Nelen V, Kwiatkowski M, Lujan M, Lilja H, Zappa M : Screening and prostate-cancer mortality in a randomized European study. The New England journal of medicine 2009, 360(13):1320–1328. doi:10.1056/NEJMoa0810084 19297566

[140] Ankerst DP, Hoefler J, Bock S, Goodman PJ, Vickers A, Hernandez J, Sokoll LJ, Sanda MG, Wei JT, Leach RJ : Prostate Cancer Prevention Trial risk calculator 2.0 for the prediction of low- vs high-grade prostate cancer. Urology 2014, 83(6):1362–1367. doi:10.1016/j.urolo-gy.2014.02.035. 24862395 PMC4035700

[141] Punnen S, Pavan N, Parekh DJ: Finding the Wolf in Sheep’s Clothing: The 4Kscore Is a Novel Blood Test That Can Accurately Identify the Risk of Aggressive Prostate Cancer. Reviews in Urology 2015, 17(1):3–13. 26028995 PMC4444768

[142] Loeb S, Catalona WJ: The Prostate Health Index: a new test for the detection of prostate cancer. Therapeutic advances in urology 2014, 6(2):74–77. doi:10.1177/1756287213513488 24688603 PMC3943368

[143] Hessels D, Schalken JA: The use of PCA3 in the diagnosis of prostate cancer. Nature Reviews Urology 2009, 6(5):255–261. doi:10.1038/nrurol.2009.40 19424173

[144] Sommariva S, Tarricone R, Lazzeri M, Ricciardi W, Montorsi F: Prognostic Value of the Cell Cycle Progression Score in Patients with Prostate Cancer: A Systematic Review and Me- ta-analysis. European Urology 2016, 69(1):107–115. doi:10.1016/j.eururo.2014.11.038 25481455

[145] Klein EA, Cooperberg MR, Magi-Galluzzi C, Simko JP, Falzarano SM, Maddala T, Chan JM, Li J, Cowan JE, Tsiatis AC : A 17-gene assay to predict prostate cancer aggressiveness in the context of Gleason grade heterogeneity, tumor multifocality, and biopsy undersampling. European Urology 2014, 66(3):550–560. doi:10.1016/j.eururo.2014.05.004 24836057

[146] Erho N, Crisan A, Vergara IA, Mitra AP, Gh-adessi M, Buerki C, Bergstralh EJ, Kollmeyer T, Fink S, Haddad Z : Discovery and validation of a prostate cancer genomic classifier that predicts early metastasis following radical prostatectomy. PloS one 2013, 8(6):e66855. doi:10.1371/journal.pone.0066855. 23826159 PMC3691249

[147] Li R, Ravizzini GC, Gorin MA, Maurer T, Eiber M, Cooperberg MR, Alemozzaffar M, Tollefson MK, Delacroix SE, Chapin BF: The use of PET/CT in prostate cancer. Prostate cancer and prostatic diseases 2018, 21(1):4–21. doi:10.1038/s41391-017-0007-8 29230009

[148] Lindenberg ML, Turkbey B, Mena E, Choyke PL: Imaging Locally Advanced, Recurrent, and Metastatic Prostate Cancer: A Review. JAMA Oncology 2017, 3(10):1415–1422. doi:10.1001/jamaoncol.2016.5840 28097325

[149] Reske SN, Blumstein NM, Neumaier B, Gottfried HW, Finsterbusch F, Kocot D, Möller P, Glatting G, Perner S: Imaging prostate cancer with 11C-choline PET/CT. Journal of nuclear medicine: official publication, Society of Nuclear Medicine 2006, 47(8):1249–1254. 16883001

[150] Calais J, Ceci F, Eiber M, Hope TA, Hofman MS, Rischpler C, Bach-Gansmo T, Nanni C, Savir-Baruch B, Elashoff D : (18)F-fluciclovine PET-CT and (68)Ga-PSMA-11 PET-CT in patients with early biochemical recurrence after prostatectomy: a prospective, single-centre, single-arm, comparative imaging trial. The Lancet Oncology 2019, 20(9):1286–1294. doi:10.1016/s1470-2045(19)30415-2. 31375469 PMC7469487

[151] Kulshrestha RK, Vinjamuri S, England A, Nightingale J, Hogg P: The Role of 18F-Sodium Fluoride PET/CT Bone Scans in the Diagnosis of Metastatic Bone Disease from Breast and Prostate Cancer. Journal of Nuclear Medicine Technology 2016, 44(4):217–222. doi:10.2967/jnmt.116.176859 27634981

[152] Afshar-Oromieh A, Avtzi E, Giesel FL, Hol-land-Letz T, Linhart HG, Eder M, Eisenhut M, Boxler S, Hadaschik BA, Kratochwil C : The diagnostic value of PET/CT imaging with the (68)Ga-labelled PSMA ligand HBED-CC in the diagnosis of recurrent prostate cancer. European journal of nuclear medicine and molecular imaging 2015, 42(2):197–209. doi:10.1007/s00259-014-2949-6. 25411132 PMC4315487

[153] Riihimäki M, Thomsen H, Brandt A, Sundquist J, Hemminki K: What do prostate cancer patients die of? The oncologist 2011, 16(2):175–181. doi:10.1634/theoncologist.2010-0338. 21257717 PMC3228081

[154] Wilt TJ, Brawer MK, Jones KM, Barry MJ, Aronson WJ, Fox S, Gingrich JR, Wei JT, Gil-hooly P, Grob BM : Radical prostatectomy versus observation for localized prostate cancer. The New England journal of medicine 2012, 367(3):203–213. doi:10.1056/NEJMoa1113162. 22808955 PMC3429335

[155] Bill-Axelson A, Holmberg L, Garmo H, Taari K, Busch C, Nordling S, Häggman M, Andersson SO, Andrén O, Steineck G : Radical Prostatectomy or Watchful Waiting in Prostate Cancer - 29-Year Follow-up. The New England journal of medicine 2018, 379(24):2319–2329. doi:10.1056/NEJMoa1807801 30575473

[156] Daskivich TJ, Fan KH, Koyama T, Albertsen PC, Goodman M, Hamilton AS, Hoffman RM, Stanford JL, Stroup AM, Litwin MS : Effect of age, tumor risk, and comorbidity on competing risks for survival in a U.S. population-based cohort of men with prostate cancer. Annals of internal medicine 2013, 158(10):709–717. doi:10.7326/0003-4819-158-10-201305210-00005. 23689764 PMC3760479

[157] Briganti A, Spahn M, Joniau S, Gontero P, Bianchi M, Kneitz B, Chun FK, Sun M, Graefen M, Abdollah F : Impact of age and comorbidities on long-term survival of patients with high-risk prostate cancer treated with radical prostatectomy: a multi-institutional competing-risks analysis. European Urology 2013, 63(4):693–701. doi:10.1016/j.eururo.2012.08.054 22959192

[158] Wang EH, Gross CP, Tilburt JC, Yu JB, Nguyen PL, Smaldone MC, Shah ND, Abouassally R, Sun M, Kim SP: Shared decision making and use of decision AIDS for localized prostate cancer: perceptions from radiation oncologists and urologists. JAMA internal medicine 2015, 175(5):792–799. doi:10.1001/jamaint-ernmed.2015.63 25751604

[159] Kweldam CF, Wildhagen MF, Steyerberg EW, Bangma CH, van der Kwast TH, van Leenders GJ: Cribriform growth is highly predictive for postoperative metastasis and disease-specific death in Gleason score 7 prostate cancer. Modern pathology: an official journal of the United States and Canadian Academy of Pathology, Inc 2015, 28(3):457–464. doi:10.1038/mod-pathol.2014.116 25189638

[160] Kweldam CF, Kümmerlin IP, Nieboer D, Verhoef EI, Steyerberg EW, van der Kwast TH, Roobol MJ, van Leenders GJ: Disease-specific survival of patients with invasive cribriform and intraductal prostate cancer at diagnostic biopsy. Modern pathology: an official journal of the United States and Canadian Academy of Pathology, Inc 2016, 29(6):630–636. doi:10.1038/modpathol.2016.49 26939875

[161] Kweldam CF, Kümmerlin IP, Nieboer D, Steyerberg EW, Bangma CH, Incrocci L, van der Kwast TH, Roobol MJ, van Leenders GJ: Presence of invasive cribriform or intraductal growth at biopsy outperforms percentage grade 4 in predicting outcome of Gleason score 3+4=7 prostate cancer. Modern pathology: an official journal of the United States and Canadian Academy of Pathology, Inc., 2017, 30(8):1126–1132. doi:10.1038/modpathol.2017.29 28530220

[162] Epstein JI, Zelefsky MJ, Sjoberg DD, Nelson JB, Egevad L, Magi-Galluzzi C, Vickers AJ, Parwani AV, Reuter VE, Fine SW : A Contemporary Prostate Cancer Grading System: A Validated Alternative to the Gleason Score. European urology 2016, 69(3):428–435. doi:10.1016/j.eururo.2015.06.046. 26166626 PMC5002992

[163] Kane CJ, Eggener SE, Shindel AW, Andriole GL: Variability in Outcomes for Patients with Intermediate-risk Prostate Cancer (Gleason Score 7, International Society of Urological Pathology Gleason Group 2-3) and Implications for Risk Stratification: A Systematic Reviews. European Urology Focus 2017, 3(4-5):487–497. doi:10.1016/j.euf.2016.10.010 28753804

[164] Musunuru HB, Yamamoto T, Klotz L, Ghanem G, Mamedov A, Sethukavalan P, Jethava V, Jain S, Zhang L, Vesprini D : Active Surveillance for Intermediate Risk Prostate Cancer: Survival Outcomes in the Sunnybrook Experience. The Journal of Urology 2016, 196(6):1651–1658. doi:10.1016/j.juro.2016.06.102 27569437

[165] Klotz L: Active surveillance in intermediate-risk prostate cancer. BJU international 2020, 125(3):346–354. doi:10.1111/bju.14935 31647166

[166] Zaffuto E, Gandaglia G, Fossati N, Dell’Oglio P, Moschini M, Cucchiara V, Suardi N, Mirone V, Bandini M, Shariat SF : Early Postoperative Radiotherapy is Associated with Worse Functional Outcomes in Patients with Prostate Cancer. The Journal of Urology 2017, 197(3 Pt 1):669–675. doi:10.1016/j.juro.2016.09.079 27670915

[167] Vale CL, Fisher D, Kneebone A, Parker C, Pearse M, Richaud P, Sargos P, Sydes MR, Brawley C, Brihoum M : Adjuvant or early salvage radiotherapy for the treatment of localised and locally advanced prostate cancer: a prospectively planned systematic review and meta-analysis of aggregate data. Lancet (London, England) 2020, 396(10260):1422–1431. doi:10.1016/s0140-6736(20)31952-8. 33002431 PMC7611137

[168] Stephenson AJ, Shariat SF, Zelefsky MJ, Kattan MW, Butler EB, Teh BS, Klein EA, Kupelian PA, Roehrborn CG, Pistenmaa DA : Salvage radiotherapy for recurrent prostate cancer after radical prostatectomy. Jama 2004, 291(11):1325–1332. doi:10.1001/jama.291.11.1325 15026399

[169] Trock BJ, Han M, Freedland SJ, Humphreys EB, DeWeese TL, Partin AW, Walsh PC: Prostate cancer-specific survival following salvage radiotherapy vs observation in men with biochemical recurrence after radical prostatectomy. Jama 2008, 299(23):2760–2769. doi:10.1001/jama.299.23.2760. 18560003 PMC3076799

[170] Thompson IM, Valicenti RK, Albertsen P, Davis BJ, Goldenberg SL, Hahn C, Klein E, Michalski J, Roach M, Sartor O : Adjuvant and salvage radiotherapy after prostatectomy: AUA/ ASTRO Guideline. The Journal of Urology 2013, 190(2):441–449. doi:10.1016/j.juro.2013.05.032 23707439

[171] Hamdy FC, Donovan JL, Lane JA, Mason M, Metcalfe C, Holding P, Davis M, Peters TJ, Turner EL, Martin RM : 10-Year Outcomes after Monitoring, Surgery, or Radiotherapy for Localized Prostate Cancer. The New England journal of medicine 2016, 375(15):1415–1424. doi:10.1056/NEJMoa1606220 27626136

[172] Donovan JL, Hamdy FC, Lane JA, Mason M, Metcalfe C, Walsh E, Blazeby JM, Peters TJ, Holding P, Bonnington S : Patient-Reported Outcomes after Monitoring, Surgery, or Radiotherapy for Prostate Cancer. The New England journal of medicine 2016, 375(15):1425–1437. doi:10.1056/NEJMoa1606221. 27626365 PMC5134995

[173] Tay KJ, Scheltema MJ, Ahmed HU, Barret E, Coleman JA, Dominguez-Escrig J, Ghai S, Huang J, Jones JS, Klotz LH : Patient selection for prostate focal therapy in the era of active surveillance: an International Delphi Consensus Project. Prostate cancer and prostatic diseases 2017, 20(3):294–299. doi:10.1038/pcan.2017.8 28349978

[174] Ahmed HU, Hindley RG, Dickinson L, Freeman A, Kirkham AP, Sahu M, Scott R, Allen C, Van der Meulen J, Emberton M: Focal therapy for localised unifocal and multifocal prostate cancer: a prospective development study. The Lancet Oncology 2012, 13(6):622–632. doi:10.1016/s1470-2045(12)70121-3. 22512844 PMC3366323

[175] Valerio M, Ahmed HU, Emberton M, Law-rentschuk N, Lazzeri M, Montironi R, Nguyen PL, Trachtenberg J, Polascik TJ: The role of focal therapy in the management of localised prostate cancer: a systematic review. European urology 2014, 66(4):732–751. doi:10.1016/j.euru-ro.2013.05.048. 23769825 PMC4179888

[176] Donaldson IA, Alonzi R, Barratt D, Barret E, Berge V, Bott S, Bottomley D, Eggener S, Ehdaie B, Emberton M : Focal therapy: patients, interventions, and outcomes--a report from a consensus meeting. European Urology 2015, 67(4):771–777. doi:10.1016/j.euru-ro.2014.09.018. 25281389 PMC4410301

[177] Cornford P, Bellmunt J, Bolla M, Briers E, De Santis M, Gross T, Henry AM, Joniau S, Lam TB, Mason MD : EAU-ESTRO-SI-OG Guidelines on Prostate Cancer. Part II: Treatment of Relapsing, Metastatic, and Castration-Resistant Prostate Cancer. European Urology 2017, 71(4):630–642. doi:10.1016/j.eururo.2016.08.002 27591931

[178] Taylor LG, Canfield SE, Du XL: Review of major adverse effects of androgen-deprivation therapy in men with prostate cancer. Cancer 2009, 115(11):2388–2399. doi:10.1002/cncr.24283 19399748

[179] Nguyen PL, Alibhai SM, Basaria S, D’Amico AV, Kantoff PW, Keating NL, Penson DF, Rosario DJ, Tombal B, Smith MR: Adverse effects of androgen deprivation therapy and strategies to mitigate them. European Urology 2015, 67(5):825–836. doi:10.1016/j.euru-ro.2014.07.010 25097095

[180] Saigal CS, Gore JL, Krupski TL, Hanley J, Schonlau M, Litwin MS: Androgen deprivation therapy increases cardiovascular morbidity in men with prostate cancer. Cancer 2007, 110(7):1493–1500. doi:10.1002/cncr.22933 17657815

[181] Alibhai SM, Duong-Hua M, Sutradhar R, Fleshner NE, Warde P, Cheung AM, Paszat LF: Impact of androgen deprivation therapy on cardiovascular disease and diabetes. Journal of clinical oncology: official journal of the American Society of Clinical Oncology 2009, 27(21):3452–3458. doi:10.1200/jco.2008.20.0923. 19506162 PMC5233456

[182] Nguyen PL, Je Y, Schutz FA, Hoffman KE, Hu JC, Parekh A, Beckman JA, Choueiri TK: Association of androgen deprivation therapy with cardiovascular death in patients with prostate cancer: a meta-analysis of randomized trials. Jama 2011, 306(21):2359–2366. doi:10.1001/jama.2011.1745 22147380

[183] Bosco C, Bosnyak Z, Malmberg A, Adolfsson J, Keating NL, Van Hemelrijck M: Quantifying observational evidence for risk of fatal and nonfatal cardiovascular disease following androgen deprivation therapy for prostate cancer: a me-ta-analysis. European Urology 2015, 68(3):386–396. doi:10.1016/j.eururo.2014.11.039 25484142

[184] Nelson CJ, Lee JS, Gamboa MC, Roth AJ: Cognitive effects of hormone therapy in men with prostate cancer: a review. Cancer 2008, 113(5):1097–1106. doi:10.1002/cncr.23658. 18666210 PMC4333639

[185] Gonzalez BD, Jim HS, Booth-Jones M, Small BJ, Sutton SK, Lin HY, Park JY, Spiess PE, Fishman MN, Jacobsen PB: Course and Predictors of Cognitive Function in Patients With Prostate Cancer Receiving Androgen-Deprivation Therapy: A Controlled Comparison. Journal of clinical oncology: official journal of the American Society of Clinical Oncology 2015, 33(18):2021–2027. doi:10.1200/jco.2014.60.1963. 25964245 PMC4461804

[186] Perera M, Roberts MJ, Klotz L, Higano CS, Papa N, Sengupta S, Bolton D, Lawrentschuk N: Intermittent versus continuous androgen deprivation therapy for advanced prostate cancer. Nature Reviews Urology 2020, 17(8):469–481. doi:10.1038/s41585-020-0335-7 32606361

[187] Corcoran C, Rani S, O’Brien K, O’Neill A, Prencipe M, Sheikh R, Webb G, McDermott R, Watson W, Crown J : Docetaxel-resistance in prostate cancer: evaluating associated phenotypic changes and potential for resistance transfer via exosomes. PloS one 2012, 7(12):e50999. doi:10.1371/journal.pone.0050999. 23251413 PMC3519481

[188] de Bono JS, Oudard S, Ozguroglu M, Hansen S, Machiels JP, Kocak I, Gravis G, Bodrogi I, Mackenzie MJ, Shen L : Prednisone plus cabazitaxel or mitoxantrone for metastatic castration-resistant prostate cancer progressing after docetaxel treatment: a randomised open-label trial. Lancet (London, England) 2010, 376(9747):1147–1154. doi:10.1016/s0140-6736(10)61389-x 20888992

[189] Oudard S, Fizazi K, Sengeløv L, Daugaard G, Saad F, Hansen S, Hjälm-Eriksson M, Jassem J, Thiery-Vuillemin A, Caffo O : Cabazitaxel Versus Docetaxel As First-Line Therapy for Patients With Metastatic Castration-Resistant Prostate Cancer: A Randomized Phase III Tri-al-FIRSTANA. Journal of clinical oncology: official journal of the American Society of Clinical Oncology 2017, 35(28):3189–3197. doi:10.1200/jco.2016.72.1068 28753384

[190] Massard C, Fizazi K: Targeting continued androgen receptor signaling in prostate cancer. Clinical cancer research: an official journal of the American Association for Cancer Research 2011, 17(12):3876–3883. doi:10.1158/1078-0432.Ccr-10-2815 21680543

[191] Stanbrough M, Bubley GJ, Ross K, Golub TR, Rubin MA, Penning TM, Febbo PG, Balk SP: Increased expression of genes converting adrenal androgens to testosterone in androgen-independent prostate cancer. Cancer research 2006, 66(5):2815–2825. doi:10.1158/0008-5472.Can-05-4000 16510604

[192] de Bono JS, Logothetis CJ, Molina A, Fizazi K, North S, Chu L, Chi KN, Jones RJ, Goodman OB Jr., , Saad F : Abiraterone and increased survival in metastatic prostate cancer. The New England journal of medicine 2011, 364(21):1995–2005. doi:10.1056/NEJ-Moa1014618. 21612468 PMC3471149

[193] Fizazi K, Scher HI, Molina A, Logothetis CJ, Chi KN, Jones RJ, Staffurth JN, North S, Vogelzang NJ, Saad F : Abiraterone acetate for treatment of metastatic castration-resistant prostate cancer: final overall survival analysis of the COU-AA-301 randomised, double-blind, placebo-controlled phase 3 study. The Lancet Oncology 2012, 13(10):983–992. doi:10.1016/s1470-2045(12)70379-0 22995653

[194] Chen CD, Welsbie DS, Tran C, Baek SH, Chen R, Vessella R, Rosenfeld MG, Sawyers CL: Molecular determinants of resistance to antiandrogen therapy. Nature Medicine 2004, 10(1):33–39. doi:10.1038/nm972 14702632

[195] Chan SC, Dehm SM: Constitutive activity of the androgen receptor. Advances in pharmacology (San Diego, Calif) 2014, 70:327–366. doi:10.1016/b978-0-12-417197-8.00011-0. 24931201 PMC4277179

[196] Scher HI, Fizazi K, Saad F, Taplin ME, Sternberg CN, Miller K, de Wit R, Mulders P, Chi KN, Shore ND : Increased survival with enzalutamide in prostate cancer after chemotherapy. The New England journal of medicine 2012, 367(13):1187–1197. doi:10.1056/NEJ-Moa1207506 22894553

[197] Smith MR, Saad F, Chowdhury S, Oudard S, Hadaschik BA, Graff JN, Olmos D, Mainwaring PN, Lee JY, Uemura H : Apalutamide Treatment and Metastasis-free Survival in Prostate Cancer. The New England journal of medicine 2018, 378(15):1408–1418. doi:10.1056/NEJ-Moa1715546 29420164

[198] Fizazi K, Shore N, Tammela TL, Ulys A, Vjaters E, Polyakov S, Jievaltas M, Luz M, Alekseev B, Kuss I : Darolutamide in Nonmetastatic, Castration-Resistant Prostate Cancer. The New England journal of medicine 2019, 380(13):1235–1246. doi:10.1056/NEJMoa1815671 30763142

[199] Pezaro CJ, Omlin AG, Altavilla A, Lorente D, Ferraldeschi R, Bianchini D, Dearnaley D, Parker C, de Bono JS, Attard G: Activity of cabazitaxel in castration-resistant prostate cancer progressing after docetaxel and next-generation endocrine agents. European Urology 2014, 66(3):459–465. doi:10.1016/j.eururo.2013.11.044 24411987

[200] de Wit R, de Bono J, Sternberg CN, Fizazi K, Tombal B, Wülfing C, Kramer G, Eymard JC, Bamias A, Carles J : Cabazitaxel versus Abiraterone or Enzalutamide in Metastatic Prostate Cancer. The New England journal of medicine 2019, 381(26):2506–2518. doi:10.1056/NEJMoa1911206 31566937

[201] Gravis G, Boher JM, Joly F, Soulié M, Al-biges L, Priou F, Latorzeff I, Delva R, Krakowski I, Laguerre B : Androgen Deprivation Therapy (ADT) Plus Docetaxel Versus ADT Alone in Metastatic Non castrate Prostate Cancer: Impact of Metastatic Burden and Long-term Survival Analysis of the Randomized Phase 3 GETUG-AFU15 Trial. European urology 2016, 70(2):256–262. doi:10.1016/j.euru-ro.2015.11.005 26610858

[202] Vale CL, Burdett S, Rydzewska LHM, Albiges L, Clarke NW, Fisher D, Fizazi K, Gravis G, James ND, Mason MD : Addition of docetaxel or bisphosphonates to standard of care in men with localised or metastatic, hormone-sensitive prostate cancer: a systematic review and meta-analyses of aggregate data. The Lancet Oncology 2016, 17(2):243–256. doi:10.1016/s1470-2045(15)00489-1. 26718929 PMC4737894

[203] Kyriakopoulos CE, Chen YH, Carducci MA, Liu G, Jarrard DF, Hahn NM, Shevrin DH, Dreicer R, Hussain M, Eisenberger M : Chemohormonal Therapy in Metastatic Hormone-Sensitive Prostate Cancer: Long-Term Survival Analysis of the Randomized Phase III E3805 CHAARTED Trial. Journal of clinical oncology: official journal of the American Society of Clinical Oncology 2018, 36(11):1080–1087. doi:10.1200/jco.2017.75.3657. 29384722 PMC5891129

[204] Fizazi K, Tran N, Fein L, Matsubara N, Rodriguez-Antolin A, Alekseev BY, Özgüroğlu M, Ye D, Feyerabend S, Protheroe A : Abiraterone plus Prednisone in Metastatic, Castration-Sensitive Prostate Cancer. The New England journal of medicine 2017, 377(4):352–360. doi:10.1056/NEJMoa1704174 28578607

[205] Rydzewska LHM, Burdett S, Vale CL, Clarke NW, Fizazi K, Kheoh T, Mason MD, Miladinovic B, James ND, Parmar MKB : Adding abiraterone to androgen deprivation therapy in men with metastatic hormone-sensitive prostate cancer: A systematic review and me-ta-analysis. European journal of cancer (Oxford, England: 1990) 2017, 84:88–101. doi:10.1016/j.ejca.2017.07.003. 28800492 PMC5630199

[206] Hoyle AP, Ali A, James ND, Cook A, Parker CC, de Bono JS, Attard G, Chowdhury S, Cross WR, Dearnaley DP : Abiraterone in “High-” and “Low-risk” Metastatic Hormone-sensitive Prostate Cancer. European Urology 2019, 76(6):719–728. doi:10.1016/j.eururo.2019.08.006 31447077

[207] Davis ID, Martin AJ, Stockler MR, Begbie S, Chi KN, Chowdhury S, Coskinas X, Frydenberg M, Hague WE, Horvath LG : Enzalutamide with Standard First-Line Therapy in Metastatic Prostate Cancer. The New England journal of medicine 2019, 381(2):121–131. doi:10.1056/NE-JMoa1903835 31157964

[208] Chi KN, Agarwal N, Bjartell A, Chung BH, Pereira de Santana Gomes AJ, Given R, Juárez Soto Á, Merseburger AS, Özgüroğlu M, Uemura H : Apalutamide for Metastatic, Castration-Sensitive Prostate Cancer. The New England journal of medicine 2019, 381(1):13–24. doi:10.1056/NEJMoa1903307 31150574

[209] Smith MR, Hussain M, Saad F, Fizazi K, Sternberg CN, Crawford ED, Kopyltsov E, Park CH, Alekseev B, Montesa-Pino Á : Darolutamide and Survival in Metastatic, Hormone-Sensitive Prostate Cancer. The New England journal of medicine 2022, 386(12):1132–1142. doi:10.1056/NEJMoa2119115 35179323 PMC9844551

[210] Hensel J, Thalmann GN: Biology of Bone Metastases in Prostate Cancer. Urology 2016, 92:6–13. doi:10.1016/j.urology.2015.12.039 26768714

[211] Smith MR, Halabi S, Ryan CJ, Hussain A, Vogelzang N, Stadler W, Hauke RJ, Monk JP, Saylor P, Bhoopalam N : Randomized controlled trial of early zoledronic acid in men with castration-sensitive prostate cancer and bone metastases: results of CALGB 90202 (alliance). Journal of clinical oncology: official journal of the American Society of Clinical Oncology 2014, 32(11):1143–1150. doi:10.1200/jco.2013.51.6500: found at the end of this article. 24590644 PMC3970172

[212] Fizazi K, Carducci M, Smith M, Damião R, Brown J, Karsh L, Milecki P, Shore N, Rader M, Wang H : Denosumab versus zoledronic acid for treatment of bone metastases in men with castration-resistant prostate cancer: a randomised, double-blind study. Lancet (London, England) 2011, 377(9768):813–822. doi:10.1016/s0140-6736(10)62344-6. 21353695 PMC3090685

[213] Smith MR, Saad F, Coleman R, Shore N, Fizazi K, Tombal B, Miller K, Sieber P, Karsh L, Damião R : Denosumab and bone-metastasis-free survival in men with castration-resistant prostate cancer: results of a phase 3, randomised, placebo-controlled trial. Lancet (London, England) 2012, 379(9810):39–46. doi:10.1016/s0140-6736(11)61226-9. 22093187 PMC3671878

[214] Parker C, Nilsson S, Heinrich D, Helle SI, O’Sullivan JM, Fosså SD, Chodacki A, Wiechno P, Logue J, Seke M : Alpha emitter radium-223 and survival in metastatic prostate cancer. The New England journal of medicine 2013, 369(3):213–223. doi:10.1056/NEJMoa1213755 23863050

[215] Gulley JL, Borre M, Vogelzang NJ, Ng S, Agarwal N, Parker CC, Pook DW, Rathenborg P, Flaig TW, Carles J : Phase III Trial of PROSTVAC in Asymptomatic or Minimally Symptomatic Metastatic Castration-Resistant Prostate Cancer. Journal of clinical oncology: official journal of the American Society of Clinical Oncology 2019, 37(13):1051–1061. doi:10.1200/jco.18.02031. 30817251 PMC6494360

[216] Kantoff PW, Schuetz TJ, Blumenstein BA, Glode LM, Bilhartz DL, Wyand M, Manson K, Panicali DL, Laus R, Schlom J : Overall survival analysis of a phase II randomized controlled trial of a Poxviral-based PSA-targeted immunotherapy in metastatic castration-resistant prostate cancer. Journal of clinical oncology: official journal of the American Society of Clinical Oncology 2010, 28(7):1099–1105. doi:10.1200/jco.2009.25.0597: found at the end of this article. 20100959 PMC2834462

[217] Kantoff PW, Higano CS, Shore ND, Berger ER, Small EJ, Penson DF, Redfern CH, Ferrari AC, Dreicer R, Sims RB : Sipuleucel-T immunotherapy for castration-resistant prostate cancer. The New England journal of medicine 2010, 363(5):411–422. doi:10.1056/NEJMoa1001294 20818862

[218] Hofman MS, Violet J, Hicks RJ, Ferdinandus J, Thang SP, Akhurst T, Iravani A, Kong G, Ravi Kumar A, Murphy DG : [(177)Lu]-PS-MA-617 radionuclide treatment in patients with metastatic castration-resistant prostate cancer (LuPSMA trial): a single-centre, single-arm, phase 2 study. The Lancet Oncology 2018, 19(6):825–833. doi:10.1016/s1470-2045(18)30198-0 29752180

[219] Sartor O, de Bono J, Chi KN, Fizazi K, Herrmann K, Rahbar K, Tagawa ST, Nordquist LT, Vaishampayan N, El-Haddad G : Lutetium-177-PSMA-617 for Metastatic Castration-Resistant Prostate Cancer. The New England journal of medicine 2021, 385(12):1091–1103. doi:10.1056/NEJMoa2107322. 34161051 PMC8446332

[220] Barata P, Agarwal N, Nussenzveig R, Gerendash B, Jaeger E, Hatton W, Ledet E, Lewis B, Layton J, Babiker H : Clinical activity of pembrolizumab in metastatic prostate cancer with microsatellite instability high (MSI-H) detected by circulating tumor DNA. Journal for immunotherapy of cancer 2020, 8(2). doi:10.1136/jitc-2020-001065. PMC7422632 32788235

[221] Tucker MD, Zhu J, Marin D, Gupta RT, Gupta S, Berry WR, Ramalingam S, Zhang T, Harrison M, Wu Y : Pembrolizumab in men with heavily treated metastatic castrate-resistant prostate cancer. Cancer medicine 2019, 8(10):4644–4655. doi:10.1002/cam4.2375. 31270961 PMC6712455

[222] Antonarakis ES, Piulats JM, Gross-Goupil M, Goh J, Ojamaa K, Hoimes CJ, Vaishampayan U, Berger R, Sezer A, Alanko T : Pembrolizumab for Treatment-Refractory Metastatic Castration-Resistant Prostate Cancer: Multicohort, Open-Label Phase II KEYNOTE-199 Study. Journal of clinical oncology: official journal of the American Society of Clinical Oncology 2020, 38(5):395–405. doi:10.1200/jco.19.01638. 31774688 PMC7186583

[223] Graff JN, Beer TM, Alumkal JJ, Slottke RE, Redmond WL, Thomas GV, Thompson RF, Wood MA, Koguchi Y, Chen Y : A phase II single-arm study of pembrolizumab with enzalutamide in men with metastatic castration-resistant prostate cancer progressing on enzalutamide alone. Journal for immunotherapy of cancer 2020, 8(2). doi:10.1136/jitc-2020-000642. PMC7333874 32616555

[224] Appleman LJ, Kolinsky MP, Berry WR, Retz M, Mourey L, Piulats JM, Romano E, Gravis G, Gurney H, Bono JSD : KEYNOTE-365 cohort B: Pembrolizumab (pembro) plus docetaxel and prednisone in abiraterone (abi) or enzalutamide (enza)–pretreated patients with metastatic castration-resistant prostate cancer (mCRPC)—New data after an additional 1 year of follow-up. 2021, 39(6_suppl):10–10. doi:10.1200/JCO.2021.39.6_suppl.10

[225] Berry WR, Fong PCC, Piulats JM, Appleman LJ, Conter HJ, Feyerabend S, Shore ND, Gravis G, Laguerre B, Gurney H : KEYNOTE-365 cohort C updated results: Pembrolizumab (pembro) plus enzalutamide (enza) in abiraterone (abi)-pretreated patients (pts) with metastatic castrate-resistant prostate cancer (mCRPC). 2020, 38(6_suppl):102–102. doi:10.1200/JCO.2020.38.6_suppl.102

[226] Wu YM, Cieślik M, Lonigro RJ, Vats P, Reimers MA, Cao X, Ning Y, Wang L, Kunju LP, de Sarkar N : Inactivation of CDK12 Delineates a Distinct Immunogenic Class of Advanced Prostate Cancer. Cell 2018, 173(7):1770–1782.e1714. doi:10.1016/j.cell.2018.04.034. 29906450 PMC6084431

[227] Abida W, Armenia J, Gopalan A, Brennan R, Walsh M, Barron D, Danila D, Rathkopf D, Morris M, Slovin S : Prospective Genomic Profiling of Prostate Cancer Across Disease States Reveals Germline and Somatic Alterations That May Affect Clinical Decision Making. JCO precision oncology 2017, 2017. doi:10.1200/po.17.00029. PMC5558263 28825054

[228] Abida W, Cyrta J, Heller G, Prandi D, Armenia J, Coleman I, Cieslik M, Benelli M, Robinson D, Van Allen EM : Genomic correlates of clinical outcome in advanced prostate cancer. Proceedings of the National Academy of Sciences of the United States of America 2019, 116(23):11428–11436. doi:10.1073/pnas.1902651116. 31061129 PMC6561293

[229] Abida W, Patnaik A, Campbell D, Shapiro J, Bryce AH, McDermott R, Sautois B, Vogelzang NJ, Bambury RM, Voog E : Rucaparib in Men With Metastatic Castration-Resistant Prostate Cancer Harboring a BRCA1 or BRCA2 Gene Alteration. Journal of clinical oncology: official journal of the American Society of Clinical Oncology 2020, 38(32):3763–3772. doi:10.1200/jco.20.01035. 32795228 PMC7655021

[230] de Bono J, Mateo J, Fizazi K, Saad F, Shore N, Sandhu S, Chi KN, Sartor O, Agarwal N, Olmos D : Olaparib for Metastatic Castration-Resistant Prostate Cancer. The New England journal of medicine 2020, 382(22):2091–2102. doi:10.1056/NEJMoa1911440 32343890

[231] Aparicio AM, Harzstark AL, Corn PG, Wen S, Araujo JC, Tu SM, Pagliaro LC, Kim J, Millikan RE, Ryan C : Platinum-based chemotherapy for variant castrate-resistant prostate cancer. Clinical cancer research: an official journal of the American Association for Cancer Research 2013, 19(13):3621–3630. doi:10.1158/1078-0432.Ccr-12-3791. 23649003 PMC3699964

[232] Ciriello G, Miller ML, Aksoy BA, Senbabaoglu Y, Schultz N, Sander C: Emerging landscape of oncogenic signatures across human cancers. Nature Genetics 2013, 45(10):1127–1133. doi:10.1038/ng.2762. 24071851 PMC4320046

[233] Baca SC, Prandi D, Lawrence MS, Mosquera JM, Romanel A, Drier Y, Park K, Kitabayashi N, MacDonald TY, Ghandi M : Punctuated evolution of prostate cancer genomes. Cell 2013, 153(3):666–677. doi:10.1016/j.cell.2013.03.021. 23622249 PMC3690918

[234] Hieronymus H, Schultz N, Gopalan A, Carver BS, Chang MT, Xiao Y, Heguy A, Huberman K, Bernstein M, Assel M : Copy number alteration burden predicts prostate cancer relapse. Proceedings of the National Academy of Sciences of the United States of America 2014, 111(30):11139–11144. doi:10.1073/pnas.1411446111. 25024180 PMC4121784

[235] Jardim DL, Goodman A, de Melo Gagliato D, Kurzrock R: The Challenges of Tumor Mutational Burden as an Immunotherapy Biomarker. Cancer cell 2021, 39(2):154–173. doi:10.1016/j.ccell.2020.10.001. 33125859 PMC7878292

[236] Sharma P, Pachynski RK, Narayan V, Fléchon A, Gravis G, Galsky MD, Mahammedi H, Patnaik A, Subudhi SK, Ciprotti M : Nivolumab Plus Ipilimumab for Metastatic Castration-Resistant Prostate Cancer: Preliminary Analysis of Patients in the CheckMate 650 Trial. Cancer cell 2020, 38(4):489–499.e483. doi:10.1016/j.ccell.2020.08.007 32916128

[237] Gao J, Ward JF, Pettaway CA, Shi LZ, Subudhi SK, Vence LM, Zhao H, Chen J, Chen H, Efstathiou E : VISTA is an inhibitory immune checkpoint that is increased after ipilimumab therapy in patients with prostate cancer. Nature Medicine 2017, 23(5):551–555. doi:10.1038/nm.4308. PMC5466900 28346412

[238] Drake CG: Prostate cancer as a model for tumour immunotherapy. Nature Reviews Immunology 2010, 10(8):580–593. doi:10.1038/nri2817. PMC3082366 20651745

[239] Faget DV, Ren Q, Stewart SA: Unmasking senescence: context-dependent effects of SASP in cancer. Nature Reviews Cancer 2019, 19(8):439–453. doi:10.1038/s41568-019-0156-2 31235879

[240] Toso A, Revandkar A, Di Mitri D, Guccini I, Proietti M, Sarti M, Pinton S, Zhang J, Kalathur M, Civenni G : Enhancing chemotherapy efficacy in Pten-deficient prostate tumors by activating the senescence-associated antitumor immunity. Cell Reports 2014, 9(1):75–89. doi:10.1016/j.celrep.2014.08.044 25263564

[241] Di Mitri D, Toso A, Chen JJ, Sarti M, Pinton S, Jost TR, D’Antuono R, Montani E, Garcia-Escudero R, Guccini I : Tumour-infiltrating Gr-1+ myeloid cells antagonize senescence in cancer. Nature 2014, 515(7525):134–137. doi:10.1038/na-ture13638 25156255

[242] Guccini I, Revandkar A, D’Ambrosio M, Colucci M, Pasquini E, Mosole S, Troiani M, Brina D, Sheibani-Tezerji R, Elia AR : Senescence Reprogramming by TIMP1 Deficiency Promotes Prostate Cancer Metastasis. Cancer cell 2021, 39(1):68–82.e69. doi:10.1016/j.ccell.2020.10.012 33186519

[243] Demaria M, O’Leary MN, Chang J, Shao L, Liu S, Alimirah F, Koenig K, Le C, Mitin N, Deal AM : Cellular Senescence Promotes Adverse Effects of Chemotherapy and Cancer Relapse. Cancer Discovery 2017, 7(2):165–176. doi:10.1158/2159-8290.Cd-16-0241. 27979832 PMC5296251

[244] Hsu CH, Altschuler SJ, Wu LF: Patterns of Early p21 Dynamics Determine Proliferation-Senescence Cell Fate after Chemotherapy. Cell 2019, 178(2):361–373.e312. doi:10.1016/j.cell.2019.05.041. 31204100 PMC6688629

[245] Perry AS, Watson RW, Lawler M, Hollywood D: The epigenome as a therapeutic target in prostate cancer. Nature Reviews Urology 2010, 7(12):668–680. doi:10.1038/nrurol.2010.185 21060342

[246] Hanahan D: Hallmarks of Cancer: New Dimensions. Cancer Discov 2022, 12(1):31–46. doi:10.1158/2159-8290.CD-21-1059 35022204

[247] Partin AW, Van Neste L, Klein EA, Marks LS, Gee JR, Troyer DA, Rieger-Christ K, Jones JS, Magi-Galluzzi C, Mangold LA : Clinical validation of an epigenetic assay to predict negative histopathological results in repeat prostate biopsies. The Journal of Urology 2014, 192(4):1081–1087. doi:10.1016/j.juro.2014.04.013. 24747657 PMC4337855

[248] Stelloo S, Nevedomskaya E, Kim Y, Schuurman K, Valle-Encinas E, Lobo J, Krijgsman O, Peeper DS, Chang SL, Feng FY : Integrative epigenetic taxonomy of primary prostate cancer. Nature Communications 2018, 9(1):4900. doi:10.1038/s41467-018-07270-2. PMC6249266 30464211

[249] Houlahan KE, Shiah YJ, Gusev A, Yuan J, Ahmed M, Shetty A, Ramanand SG, Yao CQ, Bell C, O’Connor E : Genome-wide germline correlates of the epigenetic landscape of prostate cancer. Nature Medicine 2019, 25(10):1615–1626. doi:10.1038/s41591-019-0579-z. PMC7418214 31591588

[250] Zhao SG, Chen WS, Li H, Foye A, Zhang M, Sjöström M, Aggarwal R, Playdle D, Liao A, Alumkal JJ : The DNA methylation landscape of advanced prostate cancer. Nature Genetics 2020, 52(8):778–789. doi:10.1038/s41588-020-0648-8. 32661416 PMC7454228

